# Mycorrhiza-mediated recruitment of complete denitrifying *Pseudomonas* reduces N_2_O emissions from soil

**DOI:** 10.1186/s40168-023-01466-5

**Published:** 2023-03-09

**Authors:** Xia Li, Ruotong Zhao, Dandan Li, Guangzhou Wang, Shuikuan Bei, Xiaotang Ju, Ran An, Long Li, Thomas W. Kuyper, Peter Christie, Franz S. Bender, Ciska Veen, Marcel G. A. van der Heijden, Wim H. van der Putten, Fusuo Zhang, Klaus Butterbach-Bahl, Junling Zhang

**Affiliations:** 1 College of Resources and Environmental Sciences, National Academy of Agriculture Green Development, Key Laboratory of Plant-Soil Interactions, Ministry of Education, China Agricultural University, Beijing, 100193 China; 2 College of Agronomy and Life Science, Shanxi Datong University, Datong, 037009 China; 3 College of Tropical Crops, Hainan University, Haikou, 570228 China; 4 Department of Soil Quality, Wageningen University, P.O. Box 47, Wageningen, 6700 AA The Netherlands; 5 Department of Plant and Microbial Biology, University of Zürich, Zollikerstrasse 107, CH-8008 Zürich, Switzerland; 6 Plant-Soil Interactions, Research Division Agroecology and Environment, Agroscope, Zurich, Switzerland; 7 Department of Terrestrial Ecology, Netherlands Institute of Ecology (NIOO KNAW), Wageningen, NL-6700 AB The Netherlands; 8 Karlsruhe Institute of Technology, Institute for Meteorology and Climate Research, Atmospheric Environmental Research (IMK-IFU), Kreuzeckbahnstrasse 19, 82467 Garmisch-Partenkirchen, Germany; 9 Pioneer Center Land-CRAFT, Department of Agroecology, Aarhus University, Aarhus, Denmark

**Keywords:** Arbuscular mycorrhizal fungi, N_2_O, *nosZ*, Carboxylates, *Pseudomonas*

## Abstract

**Background:**

Arbuscular mycorrhizal fungi (AMF) are key soil organisms and their extensive hyphae create a unique hyphosphere associated with microbes actively involved in N cycling. However, the underlying mechanisms how AMF and hyphae-associated microbes may cooperate to influence N_2_O emissions from “hot spot” residue patches remain unclear. Here we explored the key microbes in the hyphosphere involved in N_2_O production and consumption using amplicon and shotgun metagenomic sequencing. Chemotaxis, growth and N_2_O emissions of isolated N_2_O-reducing bacteria in response to hyphal exudates were tested using in vitro cultures and inoculation experiments.

**Results:**

AMF hyphae reduced denitrification-derived N_2_O emission (max. 63%) in C- and N-rich residue patches. AMF consistently enhanced the abundance and expression of clade I *nosZ* gene, and inconsistently increased that of *nirS* and *nirK* genes. The reduction of N_2_O emissions in the hyphosphere was linked to N_2_O-reducing *Pseudomonas* specifically enriched by AMF, concurring with the increase in the relative abundance of the key genes involved in bacterial citrate cycle. Phenotypic characterization of the isolated complete denitrifying *P. fluorescens* strain JL1 (possessing clade I *nosZ*) indicated that the decline of net N_2_O emission was a result of upregulated *nosZ* expression in *P*. *fluorescens* following hyphal exudation (e.g. carboxylates). These findings were further validated by re-inoculating sterilized residue patches with *P*. *fluorescens* and by an 11-year-long field experiment showing significant positive correlation between hyphal length density with the abundance of clade I *nosZ* gene.

**Conclusions:**

The cooperation between AMF and the N_2_O-reducing *Pseudomonas* residing on hyphae significantly reduce N_2_O emissions in the microsites. Carboxylates exuded by hyphae act as attractants in recruiting *P*. *fluorescens* and also as stimulants triggering *nosZ* gene expression. Our discovery indicates that reinforcing synergies between AMF and hyphosphere microbiome may provide unexplored opportunities to stimulate N_2_O consumption in nutrient-enriched microsites, and consequently reduce N_2_O emissions from soils. This knowledge opens novel avenues to exploit cross-kingdom microbial interactions for sustainable agriculture and for climate change mitigation.

Video Abstract

**Supplementary Information:**

The online version contains supplementary material available at 10.1186/s40168-023-01466-5.

## Introduction

Nitrous oxide (N_2_O) is a very powerful and long-lived greenhouse gas with 273 times the global warming potential of CO_2_ and is the most important ozone-depleting substance present in the atmosphere [1]. However, constraining the global atmospheric N_2_O budget remains challenging as N_2_O fluxes at the soil-atmosphere interface are highly dynamic and variable, characterized by “hot spots” and “hot moments” at microscales that are often < 1 cm^3^ in volume and associated with crop residue patches in agriculture [2]. Estimates of N_2_O emission factors of crop residues vary widely, ranging from 0.17 to 2.9% [3], depending on residue properties [4] and multiple environmental factors such as C/N ratio, soil type, water-filled pore space (WFPS) and temperature [5]. The high spatiotemporal dynamics of N_2_O fluxes are due to the complex microbial processes underlying N_2_O production and consumption, and how these are affected by other biotic and abiotic factors [6]. As such, uncovering microbial interactions at the microscale level that mediate the episodic N_2_O emissions is critical for the development of mitigation strategies.

The production of N_2_O in soils is driven mainly by microbial driven processes such as nitrification and denitrification [6]. Denitrification is regarded as the predominant N_2_O source from agricultural soils [7] including soils where crop residues are returned, as the provision of degradable organic matter stimulates microbial respiration, resulting in oxygen depletion and soil anaerobiosis [2, 5]. Denitrification is a facultative process that enables the maintenance of microbial respiration. It involves a multistep reaction catalyzed by multiple enzymes and the relevant functional genes that used to characterize microbes, as denitrifiers are highly diverse and complex. Denitrifiers can produce N_2_O using two types of dissimilatory nitrite reductase encoded by the *nirS* and *nirK* genes that catalyze the reduction of soluble NO_2_^−^ to gaseous NO, followed by rapid conversion to N_2_O as a detoxification approach [8]. Complete denitrifiers also synthesize the N_2_O reductase (nosZ) encoded by the *nosZ* gene and yield N_2_ as the end product of denitrification, which is an important biotic sink for N_2_O [9]. The nosZ protein phylogeny has two distinct groups, clade I and the newly described clade II. The clade I *nosZ*-possessing microorganisms are more likely to be complete denitrifiers, as 83% of genomes with clade I *nosZ* also possess *nirS* and/or *nirK* genes [10]. In contrast, the majority of microorganisms possessing clade II *nosZ* appear to be non-denitrifying N_2_O reducers which represent another important N_2_O sink without contributing to N_2_O production [10–12]. Hence, soil N_2_O emissions at the soil-atmosphere interface are highly dynamic, resulting from simultaneously occurring production and consumption processes. An in-depth understanding of the mechanisms by which soil microbial guilds govern the balance of these key processes is important for the development of effective N_2_O mitigation strategies.

Microbial N_2_O production and consumption in crop residue patches and surrounding soil are part of a complex suite of processes carried out by a consortium of microbiomes, including plant-associated microbes such as arbuscular mycorrhizal fungi (AMF). AMF are key organisms with a dual niche in host roots and in the bulk soil beyond the rhizosphere [13]. The extraradical fungal hyphae represent an important component and can proliferate into micropores inaccessible to plant roots and increase carbon flow into the soil [14], generating a unique microhabitat-hyphosphere, an extension of the rhizosphere where hyphae and other microbes interact intensively in a similar manner to rhizosphere hotspots [15]. This was shown by the positive feedback between AMF and hyphosphere phosphate-solubilizing bacteria in enhancing the mineralization of organic phosphorus [16, 17]. Hyphal exploration of residue patches may prime decomposition and increase nitrogen acquisition from plant residues [18]. In addition, AMF hyphae reduce N_2_O emission from residue-affected soil [19–21], which is attributable to AMF-mediated substrate changes and/or the alteration of the hyphosphere microbiome, for instance ammonium-oxidizing microbes [19, 22] or denitrifiers [20, 21, 23]. Previous studies have shown that AMF indirectly affect denitrifying microorganisms by promoting water absorption [24] or promoting soil aggregation [25]. However, direct evidence in support of AMF interacting with the hyphosphere microbiome, especially complete denitrifiers, remains ambiguous. Given that AMF receive 4–20% of total photosynthetic C from plants [26] and that hyphae form a network redistributing C into unexplored nonrhizosphere zones [14], this knowledge gap has important implications for the potential exploitation of the soil microbiome in terms of the development of suitable management practices to increase nutrient use efficiency while mitigating N_2_O emission. This is especially important in sustainable agriculture because current intensive agricultural practices result in a substantial decline in AMF diversity and abundance [27, 28] and hence hamper their potential to mitigate N_2_O emission.

Here, we have tested the underlying mechanisms responsible for AMF hyphae-mediated reduction of N_2_O emission, with special emphasis on the microbial taxa capable of complete denitrification in the hyphosphere. We first identified the major players and main pathways by integrating quantitative real-time PCR of the functional genes and amplicon sequencing based on DNA and RNA analysis. We then isolated the most responsive bacterial genus (*Pseudomonas* in this case) and tested the chemotaxis, growth and N_2_O consumption of the isolated strains in response to hyphal exudates using in vitro cultures. Subsequently, the target strain was reinoculated into sterilized residue patch soils to validate the results of the in vitro cultures. Finally, we tested whether a positive correlation between AMF abundance and *nosZ* gene copies occurred in agricultural fields. We hypothesized that bacteria colonizing hyphae, e.g. *nosZ*-type complete denitrifiers, were the major players responsible for reduced N_2_O emissions. Specifically, hyphal exudates, in particular carboxylates, elicited the recruitment of complete denitrifiers by AMF hyphae and stimulated their functions in the hyphosphere. We envisage that a mixture of hyphosphere microbes in conjunction with hyphal metabolites have great potential to reduce N_2_O emission.

## Materials and methods

### Part 1: N_2_O emissions and denitrifying communities in response to AMF hyphae

Two pot experiments (pot expts 1 and 2) were conducted to examine whether N_2_O production in faba bean (*Vicia faba* L.) residue patches declined in the presence of AMF hyphae. We also analyzed the abundance and structure of N_2_O producers and N_2_O reducers in all patches with and without AMF hyphae.

#### Pot expt 1: N_2_O emissions as affected by the presence of AMF

##### Microcosm setup

**Fig. 1:**
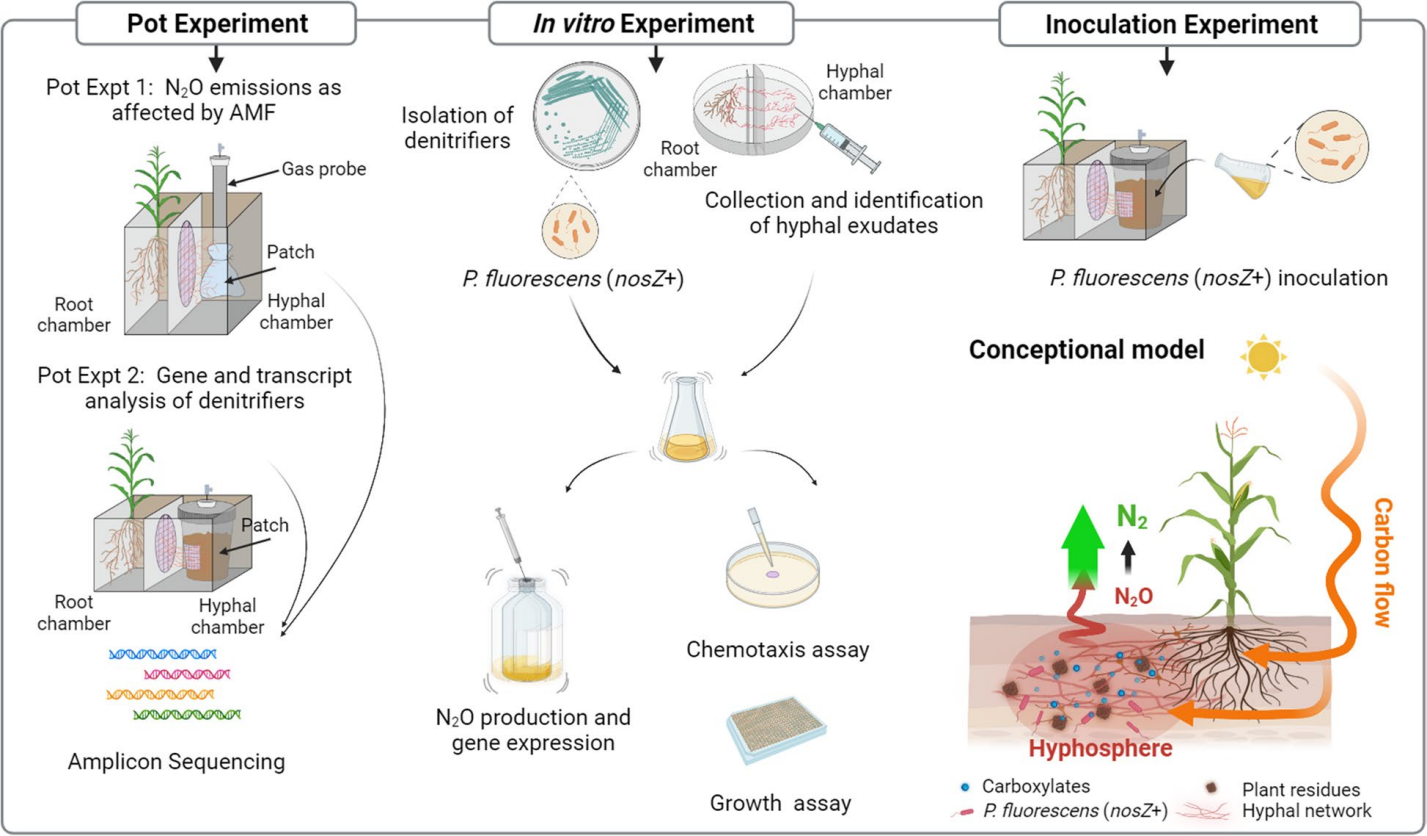
Flow chart of the experiments. The study comprised several experiments: two pot experiments, in vitro experiments, and an inoculation experiment. The two pot experiments (pot expts 1 and 2) tested whether the proliferation of AMF into microsites of residues may reduce N_2_O emissions and disassemble the regulation pathway. We also tested the abundance of the microbiome in the hyphosphere; the in vitro experiment assessed the importance of *P. fluorescens* co-colonization with AMF for reduced N_2_O emissions. We isolated denitrifiers and identified the key components of hyphal exudates. We then examined the chemotaxis, growth, N_2_O emission, and denitrifying gene expression of *P. fluorescens* in response to AMF exudates and key compounds under in vitro culture conditions; finally, the inoculation experiment validated the effects of AMF or citrate exuded by AMF on N_2_O emissions and *nosZ* gene expression of *P. fluorescens* in pot culture. A conceptual model is used to illustrate the pathways by which AMF interact with *P. fluorescens* to mitigate N_2_O production

Microcosms with two chambers, one root chamber for plant growth (3 × 10 × 15 cm^3^) and one hyphal chamber for hyphal growth (7 × 10 × 15 cm^3^), were constructed (Fig. 1). The two chambers were separated by a 30-μm or a 0.45-μm mesh that allowed or prevented AMF hyphae access to the hyphal chamber. In all cases, the roots were not allowed to access the hyphal chamber.

In each hyphal chamber, we introduced a patch with a 30-μm pore nylon mesh bag (4 × 7.5 cm^2^, 5 cm high) that could be filled with residues. A gas probe was inserted into the patch to collect gas samples to measure the N_2_O concentration as an indicator of N_2_O production in the patch (Fig. 1).

##### Plant growth substrate and AMF inoculum

The soil was collected from bare arable land at Quzhou Experimental Station (36° 52′ N, 114° 01′ E, 40 m a.s.l.) in Quzhou County, Hebei Province, North China. The soil was air-dried, sieved (< 2 mm) and mixed 1:1 (w/w) with sand to serve as a growth substrate in the pot experiment. The substrate was γ-irradiated with a maximum dose of 32 kGy to eliminate indigenous AMF. The root chamber was inoculated with the AMF *Funneliformis mosseae* (HK01). Details are shown in the Supplementary Information.

##### Gas probe and residue patches

**Gas probe**

A stainless-steel tube (15 cm high, 15 cm^3^ volume) was sealed gas-tight at its end. Two opposing windows (2 × 6 cm^2^) were opened 0.5 cm from the end of the tube. These windows were covered with a polyvinylidene difluoride (PVDF) membrane (0.22 μm) that was air-permeable but water-impermeable (Fig. 1).

**Residue patches**

Patch materials consisted of a mixture of 13 g DW (dry weight) substrate with either 2 g DW milled residues (sterilized or unsterilized) or 2 g substrate as control. The base of each gas probe was wrapped within each patch bag. Faba bean stubble was used as residue (total carbon (TC) 36.91%, total nitrogen (TN) 3.19%, C:N ratio 11.6). The stubble was oven-dried at 40 °C to maintain the root microbiome (unsterilized residue, NS) and ground in a ball mill. Portions of milled stubble were also sterilized at 121 °C for 30 min for use as sterilized residue (S). Details are shown in the Supplementary Information.

##### Experimental design

Pot expt 1 consisted of two factors: (1) patch type, sterilized or unsterilized faba bean residue patches (a mixture of 13 g substrate with either 2 g sterilized or unsterilized faba bean residue), and soil in the patch as the control (15 g substrate); and (2) AMF, presence or absence of AMF, where AMF hyphae access to the hyphal chamber was either allowed or denied. Each treatment had 8 replicates.

The root chamber contained 450 g sterilized substrate and 50 g AMF inoculum. Nutrients were supplied to the root chamber to ensure sufficient nutrients for plant growth by adding 100 mg kg^−1^ N (Ca (NO_3_)_2_·4H_2_O), 20 mg kg^−1^ P (KH_2_PO_4_), and 100 mg kg^−1^ K (K_2_SO_4_). The hyphal chamber contained 1500 g sterilized substrate only. A sterile centrifuge tube (50 mL) was placed in the center of the hyphal chamber to reserve space for the subsequent addition of the patches. Two maize (*Zea mays* L.) seeds were placed in the root chamber and thinned to one seedling after germination. The centrifuge tube was replaced with patch 30 days after maize planting. Each patch, enclosed in a 30-μm mesh bag and with a gas probe attached, was placed in the spot reserved by the centrifuge tube (Fig. 1). Then, 5 mL of microbial filtrate derived from the substrate soil was added to each patch to equalize microbial communities other than AMF [20]. The patch was then covered with 20 g substrate. Soil moisture was maintained at 60% of WFPS with deionized water by weighing the pots daily according to Li et al. [29]. The microcosm experiment was conducted in a greenhouse at China Agricultural University, Beijing, at 25–30 °C (day) /18–22 °C (night) and 60–80% relative humidity.

##### Addition of inorganic nitrogen fertilizers

Thirty-six days after patch addition (66 days after maize planting), 7 mL of 15 mM (NH_4_)_2_SO_4_ (NH_4_^+^-N treatment) or 30 mM KNO_3_ (NO_3_^−^-N treatment) solution was injected into each patch (corresponding to 0.196 mg N g^−1^ DW patch). This was performed by injecting 3.5 ml of solution twice with a 1-h interval between injections to minimize solution diffusion into the surrounding substrate. This resulted in four replicates of each nitrogen addition treatment. The gas collection details are shown in the Supplementary Information.

#### Pot expt 2: gene and transcript analysis of denitrifiers

In pot expt 2, with a duration of 55 days, we investigated whether AMF affected the abundance and expression of the *nosZ* gene in residue patches. Two factors were analyzed: (1) presence or absence of AMF and (2) harvest time, corresponding to days 24 (T1) and 34 (T2) after patch placement. Each treatment had 5 replicates.

The microcosm setup, plant growth substrate, AMF inoculum, and patches were similar to those of pot expt 1 with the following modifications. The patch effect was enlarged by modifying pot size (Fig. 1). Details are shown in the Supplementary Information. Sufficient nutrients were supplied to both chambers and patches by adding 200 mg kg^−1^ N (Ca (NO_3_)_2_·4H_2_O), 20 mg kg^−1^ P (KH_2_PO_4_), and 100 mg kg^−1^ K (K_2_SO_4_). Here, NO_3_^−^-N was added to all the chambers including the patch chambers to minimize N diffusion from the patch to the surrounding soil.

The experimental procedure was similar to that in pot expt 1. A mixture of 200 g DW substrate with 2 g DW milled unsterilized residues was placed in the patch 21 days after maize planting. Microbial filtrates were added to each patch. The N_2_O concentrations in the headspace of the bottle were monitored from day 4 after patch placement onwards at 2-day intervals until day 32 by taking 10 mL of headspace gas from the patch chamber using a syringe, at 0 and 3 h after the chamber was closed. The sampling times were 09.00 am and 12.00 am, and this time interval was selected based on the *R*^*2*^ (0.96) value found in a preliminary experiment (Table S1). The fluxes and cumulative N_2_O emissions were calculated using formulae described previously [20].

##### Plant harvest and determination of soil physicochemical properties

Pot expt 1 was harvested 6 days after the addition of inorganic nitrogen. Pot expt 2 was harvested twice, on day 24 (i.e., 45 days after maize planting) and day 34 (i.e., 55 days after maize planting) after patch placement. The details of the harvest procedure and determination of soil water content, dissolved organic carbon (DOC), total dissolved nitrogen (TDN), mineral N concentrations, hyphal length density (HLD), TC, TN, ammonium (NH_4_^+^-N), and nitrate (NO_3_^−^-N) concentrations are shown in the Supplementary Information.

##### DNA and RNA extraction, cDNA synthesis, real-time PCR, high-throughput sequencing, and shotgun metagenomic sequencing

In the two pot experiments, soil DNA and RNA were extracted from 0.50 g and 2 g fresh soil using a fast DNA SPIN Kit (MP Biomedicals, Santa Ana, CA) and an RNA PowerSoil Total RNA Isolation Kit (Mo Bio, Carlsbad, CA), respectively, according to the manufacturers’ instructions. Complementary DNA (cDNA) was synthesized from the RNA samples (1 μg) using a PrimeScript RT Reagent Kit with gDNA Eraser that includes a genomic DNA elimination reaction. Real-time quantitative PCR (qPCR) of the *nirK*, *nirS*, and *nosZ* (clade I and II) genes were conducted using QuantStudio 6 Flex (Applied Biosystems, Waltham, MA). The primers F1aCu/R3Cu, Cd3aF/ R3cd, *nosZ*2F/*nosZ*2R, and *nosZ-*II-F/*nosZ-*II-R were used, and the primer sequence and thermal conditions are shown in Table S2. The microbial communities harboring the marker genes *nirK*, *nirS*, and clade I *nosZ* were determined. Paired-end sequencing (2 × 300) was conducted through Illumina MiSeq PE high-throughput sequencing. To further explore the potential microbial functions in response to AMF, DNA samples from the second harvest in pot expt 2 were selected for shotgun metagenomics using the Illumina NovaSeq platform with a paired-end protocol [30]. The details of DNA and RNA extraction, cDNA synthesis, qPCR, high-throughput sequencing, and metagenomic sequencing are shown in the Supplementary Information.

### Part 2: in vitro experiments: chemotaxis, growth, and N_2_O production by isolated denitrifiers in response to hyphal exudates

#### Isolation, identification, and genome sequencing analysis

Denitrifier strains were isolated from patches in the presence/absence of AMF at the second harvest in pot expt 2 to examine the enriched denitrifier community in the hyphosphere. Fresh soil was vortexed and suspended in ddH_2_O. Then, 10^5^-fold dilutions of the soil suspension were spread on bromothymol blue (BTB) agar plates to isolate the denitrifiers [31]. Each sample was prepared in triplicate. The plates were incubated at 30 °C for 1–3 days. Separate blue colonies were isolated and purified by repeated streaking on BTB plates. The total bacterial DNA of each isolate was extracted from 1 mL culture suspension with a genomic DNA extraction kit (Tiangen Biotech, Beijing, China). The bacterial primers 27F/1492R were used for 16S rDNA amplification, and sequencing was performed by Tsingke Biotech, Beijing, China. The PCR thermal conditions are shown in Table S2. Following dereplication with a cut off value of 99% sequence similarity, the sequences were aligned with reference sequences in the National Center for Biotechnology Information (NCBI) GenBank database. A phylogenetic tree was then constructed by the neighbor-joining method [32] with bootstrap analysis of 1000 replicates using MEGA version 5 [33].

The bacterial primers *nosZ*1527*F*/*nosZ*1773*R* were used for *nosZ* gene amplification to examine whether the *Pseudomonas* isolates possessed the *nosZ* gene (Table S2). The target band was detected, sequenced and then identified using a BLAST search in GenBank in NCBI. Three *Pseudomonas fluorescens* isolates (JL1, JL2, and JL3) possessing the *nosZ* gene were screened. The draft genomes of the three strains were sequenced. Details are shown in the Supplementary Information.

#### Collection and analysis of hyphal exudates

An in vitro two-chamber culture was established to collect hyphal exudates [34] to examine the response of *P. fluorescens* JL1 to hyphal exudates (Fig. 1). The AMF strain used, *Rhizophagus irregularis* MUCL 43194, was grown on axenically produced transformed carrot (*Daucus carota* L.) roots. Growth and hyphal exudate harvesting were performed using a previously described protocol [17]. The collection of hyphal exudates and the analysis of sugars, carboxylates, and amino acids in the hyphal exudates are shown in the Supplementary Information. Analysis revealed concentrations of 7.16 mM TC and 2.35 mM TN in the exudate solutions. These values were used as references for subsequent experiments.

#### Serum bottle assay

A sealed serum bottle assay was conducted to examine the effects of hyphal exudates and major compounds on net N_2_O production by *P. fluorescens* JL1. Hyphal exudate was applied as one treatment. Fructose, trehalose, citrate, malate, glutamine, or glutamic acid was selected as the carbon source treatment because these compounds were detected at high concentrations in hyphal exudates. Glucose was used as the control. There were 8 treatments in total. The same liquid MSR medium [17] as that used for the collection of hyphal exudates (see Supplementary Information) was used to dissolved specific carbon source. The carbon and nitrogen contents in the medium were adjusted to the same level as those in the hyphal exudate solutions (7.16 mM C and 2.35 mM N). The hyphal exudate medium and specific compound medium were supplemented with 10% FeNaEDTA (relative to MSR medium) to ensure denitrification. The medium pH was then adjusted to 7.2, and the medium was filtered through an Acrodisc syringe filter (0.22-μm Super Membrane, Pall Corporation, Port Washington, NY) to obtain carbon-based medium (CB medium). The CB medium was supplemented with 92.84 mM glucose to reach an initial C concentration of 100 mM. NO_3_^−^-N was supplemented to reach a level of 10 mM to ensure denitrification. The pellet obtained from the centrifugation of 1 mL *P. fluorescens* JL1 suspension was re-suspended in 10 mL modified CB medium and transferred to a 120-mL anaerobic serum bottle. All serum bottles were shaken at 180 rpm and maintained at 30 °C. The gas was measured after 0.5, 1, 2, 3, 6, 8, 10, and 12 h. Each treatment was set up in triplicate. Details are shown in the Supplementary Information.

#### Assay of gene expression of denitrifiers

Gene expression of the complete denitrifier *P. fluorescens* JL1 in response to hyphal exudates was determined. Citrate and malate were selected as carbon sources based on the results from the serum bottle assay. Glucose was used as the control. The experimental design was the same as in the serum bottle assay. At 0.5, 1, 2, 3, and 6 h, total RNA were extracted and relative changes in *nirS* and *nosZ* genes normalized by the 2^−ΔΔCt^ method [35]. Details are shown in the Supplementary Information.

#### Chemotaxis assay

M8 basal medium [36] solidified with 0.3% agar was used to assay the chemotaxis of *P. fluorescens* JL1 to hyphal exudate and its main compounds. The carbon source was substituted with CB medium according to the serum bottle assay. Carbon-free CB medium was used as the control (CK), and the same in the growth assay below. M8 basal medium was autoclaved and cooled to ~ 50 °C, and CB medium was added prior to plate pouring. A final carbon concentration of 716 μΜ (according to 10% C in the hyphal exudates) was maintained in the medium. After thorough mixing, the medium was dispensed into culture plates. One microliter of *P. fluorescens* JL1 suspension (OD_600_ value 0.20, see Supplementary Information) was placed on the center of the agar layer. The plates were placed in a 28 °C incubator. The area covered by each strain, i.e., the swimming motility zone (as depicted by radial growth), was monitored and photographed after 48 h.

#### Growth assay

CB medium (see the serum bottle assay) was used to assay the growth of *P. fluorescens* JL1 in response to hyphal exudates or to its main compounds. The medium was supplemented with 300 mg L^−1^ NH_4_^+^-N (NH_4_Cl) and 10% vitamins (relative to MSR medium) to assure sufficiency for bacterial assimilation. *P. fluorescens* JL1 suspension was inoculated into 250 μL CB medium and cultured in a 10 × 10-well honeycomb microplate (initial OD_600_ value 0.05). The OD_600_ value was measured every 2 h at 30 °C for 24 h using a Bioscreen C automated microbiology growth curve analysis system (Oy Growth Curves Ab, Turku, Finland), with 4 replicates per treatment.

### Part 3: inoculation experiment

The effectiveness of *P. fluorescens* JL1 in reducing N_2_O emissions was validated by inoculating the strain into patches amended with different carbon sources in the −AMF treatment to compare their effects with the in situ hyphal exudates. The design of the microcosm, growth substrate, nutrient supplements, and patch composition were the same as in pot expt 2. The patch materials were sterilized to eliminate indigenous microorganisms after culturing in an incubator for 7 days at 25 °C and 60% of WFPS. Each patch was inoculated with *P. fluorescens* JL1 suspension at a final concentration of 10^8^ CFU bacteria g^−1^ soil. The patches were placed 21 days after maize planting. Ten days after patch placement, 2 mL carbon source dissolved in sterile H_2_O (pH 7.5) were injected slowly into the center of the patches at 18:00 on the day before the onset of gas measurement. There were four treatments in the patches: (1) absence of AMF (−AMF) with H_2_O; (2) −AMF with 7.16 mmol glucose-C kg^−1^ soil; (3) −AMF with 7.16 mmol citrate-C kg^−1^ soil; and (4) presence of AMF (+AMF), with 4 replicates per treatment. Gas was monitored every 2 days from days 2 to 24 after patch placement. Eight milliliters of headspace gas was collected from the patch chamber using a syringe 0, 1.5, and 3 h after the chamber was closed. Then, 8 mL of N_2_ was replenished quickly after every gas sampling to balance the air pressure in the patches. The sampling time was 9.00 am to 12.00 am. The soil moisture content was maintained at 60% WFPS by adjusting the weight of each pot with sterile H_2_O. RNA extraction, cDNA synthesis, and the relative change in *nirS* and *nosZ* genes were conducted and assessed as described above. The bacterial numbers in patches were counted according to the total number of colony-forming units (CFU g^−1^ soil) of bacteria [37].

### Part 4: measurements from a long-term intercropping field experiment

Samples were collected from a long-term intercropping experiment to test whether a positive correlation between AMF abundance and *nosZ* gene copies occurred in agricultural ecosystems. We selected an intercropping experiment because intercropping has been shown to increase AMF abundance compared to monocultures [38]. The long-term experiment started in 2010 at Baiyun Experimental Station, Gansu Province, Ningxia Hui Autonomous Region, Northwest China. The experiment was a split-plot completely randomized block design. Two planting patterns of faba bean monoculture and faba bean intercropped with maize at two P application rates (zero P or 40 kg P ha^−1^ year^−1^) were established, and each treatment was set up in triplicate. Details of the field management scheme have been published by Li et al. [39]. Soil samples were collected when the faba bean was at the full-bloom stage. Soil samples close to faba bean plants were collected from the top 20 cm of the soil profile using a 35-mm-diameter auger. Five soil cores were collected randomly from each plot and combined to give one composite sample per plot of monocultures or intercropping. The composite samples were sieved through a 2-mm mesh. One portion was stored at −80 °C for molecular analysis, and the remainder was air-dried for the determination of HLD. Soil DNA extraction, real-time PCR of the *nosZ* gene, and HLD were conducted and assessed as described above.

## Statistical analysis

Statistical analysis was conducted in R 4.0.3 or SPSS version 22.0. Figures were produced using the ggplot2 R package or Origin 2021. Details of the statistical analyses are shown in the Supplementary Information.

## Results

### Pot experiments: AMF reduced N_2_O emissions in residue patches

**Fig. 2:**
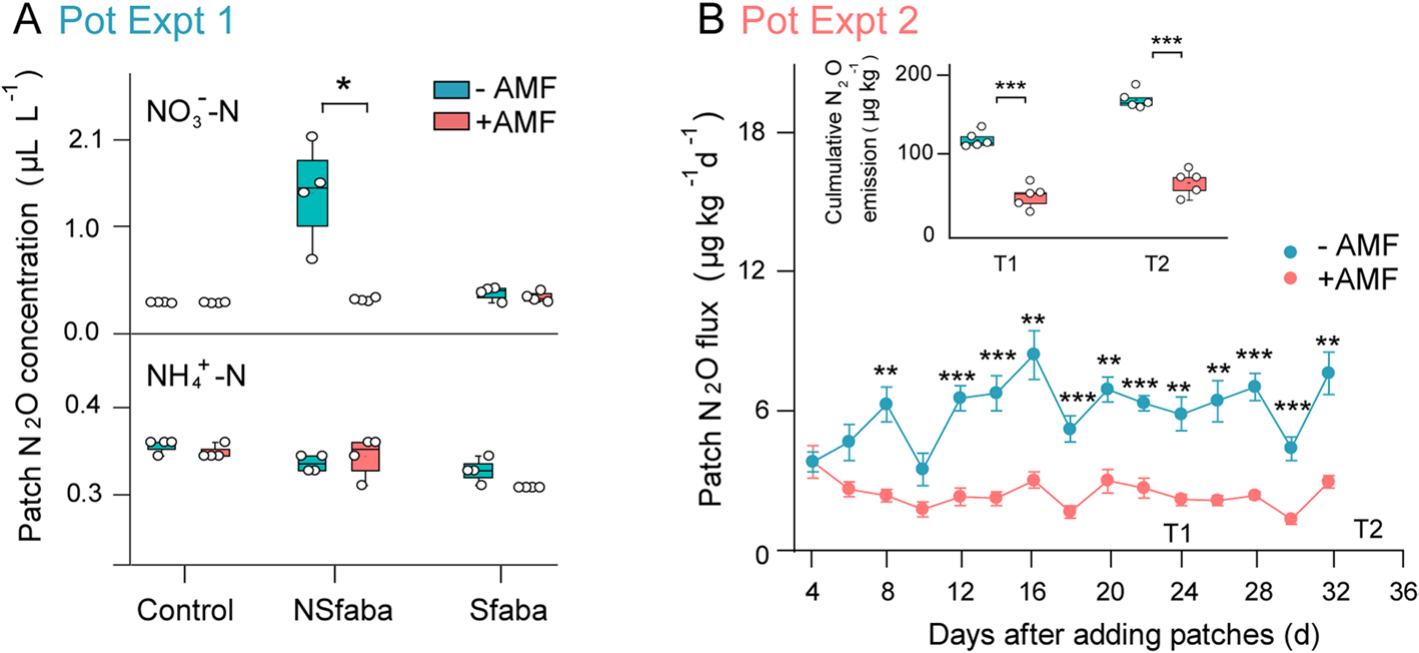
N_2_O emissions from patches in the absence (−AMF) or presence (+AMF) of AMF. **A** Pot expt 1. N_2_O concentrations from different patches under −AMF and +AMF treatments at 24 h after the addition of NO_3_^−^-N or NH_4_^+^-N (*n* = 4). Control, soil patch; NSfaba and Sfaba, patches with unsterilized (NS) or sterilized (S) faba bean residues, respectively; asterisk, significant difference between −AMF and +AMF treatments in each patch type by two-tailed unpaired *t* test under NO_3_^−^-N addition treatment (*, *P* < 0.05). **B** Pot expt 2. N_2_O flux and cumulative N_2_O emission at both harvests from patches under −AMF and +AMF treatments (*n* = 5); T1 and T2, first (day 24) and second (day 34) harvests, respectively; asterisks, significant differences between −AMF and +AMF treatments within each gas-sampling time or each harvest according to two-tailed unpaired *t* test (**, *P* < 0.01; ***, *P* < 0.001). The box plots show the 25–75th percentiles (box), the median and the mean (the band and the dot inside the box), and the minimum to maximum values excluding outliers (whiskers)

In pot expt 1 (Fig. 1), AMF hyphae grew into all patches and average HLD in patches was 5.29±0.42 m g^−1^ soil (Fig. S1A) in the +AMF treatment, which was approximately 1.9 times higher than that (1.84±0.17 m g^−1^ soil) in the −AMF treatment. High N_2_O concentrations occurred only in the unsterilized faba bean (NSfaba) patches and in the −AMF treatment 24 h after NO_3_^−^ application, but not subsequently. In contrast, the N_2_O concentration in the +AMF treatment declined significantly compared to the −AMF treatment 24 h after NO_3_^−^ application in NSfaba patches, and remained low at near-atmospheric concentrations comparable to those in the control and sterilized faba bean (Sfaba) patches (Fig. 2A and Fig. S1B). The N_2_O concentration in the NSfaba patches amended with NH_4_^+^-N was low and no significant differences between AMF treatments were observed (Fig. 2A and Fig. S1B).

In pot expt 2, the temporal dynamics of N_2_O emissions with residues amended only with NO_3_^−^-N were monitored over 1 month. The average HLD value in patches was 5.72±0.15 m g^−1^ soil in the +AMF treatment, 3.8 times higher than that (1.18±0.05 m g^−1^ soil) in the −AMF treatment (Fig. S1D). The presence of AMF hyphae significantly reduced N_2_O emission from residue patches from day 8 until the end of the experiment, with the fluxes declining by ≤70% and cumulative emissions by 63% compared to the −AMF treatment (Fig. 2B).

### Pot experiments: AMF promoted the abundance and expression of the nosZ gene and enriched N_2_O-reducing Pseudomonas in residue patches

**Fig. 3:**
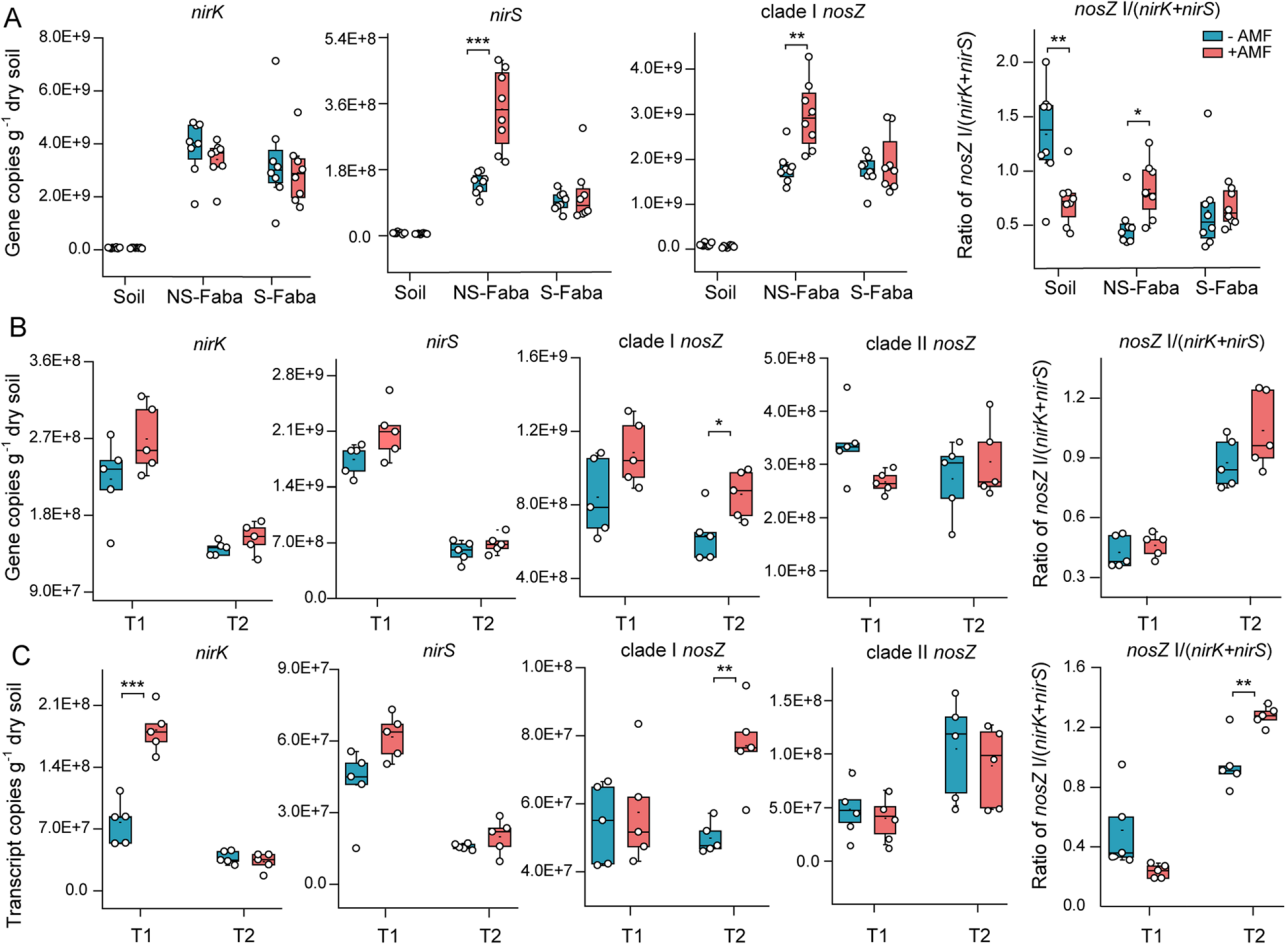
Gene and transcript copies of *nirK*, *nirS*, *nosZ* (clade I and clade II), and the *nosZ* I/(*nirK*+*nirS*) ratio in patches in the absence or presence of AMF. **A** Pot expt 1. *nirK*, *nirS*, and *nosZ* (clade I and II) gene copies and the *nosZ* I/(*nirK*+*nirS*) ratio in different patches under −AMF and +AMF treatments (*n* = 8). Control, soil patch; NSfaba and Sfaba, patches with unsterilized (NS) or sterilized (S) faba bean residues, respectively. **B** Pot expt 2. *nirK*, *nirS*, and *nosZ* (clade I and II) gene copies and the *nosZ* I/(*nirK*+*nirS*) ratio in patches under −AMF and +AMF treatments at both harvests (*n* = 5). **C** Pot expt 2. *nirK*, *nirS*, and *nosZ* (clade I and II) transcript copies and the *nosZ* I/(*nirK*+*nirS*) transcript ratio in patches under −AMF and +AMF treatments at both harvests (*n* = 5). T1 and T2, first (day 24) and second (day 34) harvests, respectively. Asterisks, significant differences between −AMF and +AMF treatments in each patch type (pot expt 1) or at each harvest (pot expt 2) by two-tailed unpaired *t* test (*, *P* < 0.05; **, *P* < 0.01; ***, *P* < 0.001)

The abundance of the key genes involved in N_2_O production (*nirK* and *nirS*) and consumption (clade I and II *nosZ*) in residue patches were determined. In pot expt 1, AMF significantly increased *nirS* and clade I *nosZ* gene copies and the ratio of *nosZ* I/(*nirK + nirS*) only in the NSfaba patches and not in the Sfaba patches or the control (soil only) (Fig. 3A). Clade I *nosZ* gene copies were negatively correlated with N_2_O concentrations in the NSfaba patches under the NO_3_^−^-N treatment (*r* = −0.78, *P* = 0.021) but not under the NH_4_^+^-N treatment (*r* = −0.23, *P* = 0.59) (Fig. S2A). Moreover, overall clade I *nosZ* gene copies were positively correlated with HLD (Fig. S2B). In pot expt 2, AMF significantly increased the *nirK* transcript copies at the first harvest and the clade I *nosZ* gene and transcript copies and transcript ratio of *nosZ* I/(*nirK* + *nirS*) at the second harvest (Fig. 3B, C). The abundance and expression of the *nirS* and clade II *nosZ* gene were not significantly affected by AMF (Fig. 3B, C). Multiple stepwise regression indicates that the variation in N_2_O emission was best explained by *nirK* gene expression at the first harvest and by clade I *nosZ* gene expression at the second harvest (Table S3). Moreover, the clade I *nosZ* gene and transcript copies were positively correlated with HLD and DOC concentrations, which were significantly increased by AMF at the second harvest (Figs. S1C and S2C, D). Based on these results, we focused on the clade I *nosZ* community in the subsequent experiment.

**Fig. 4:**
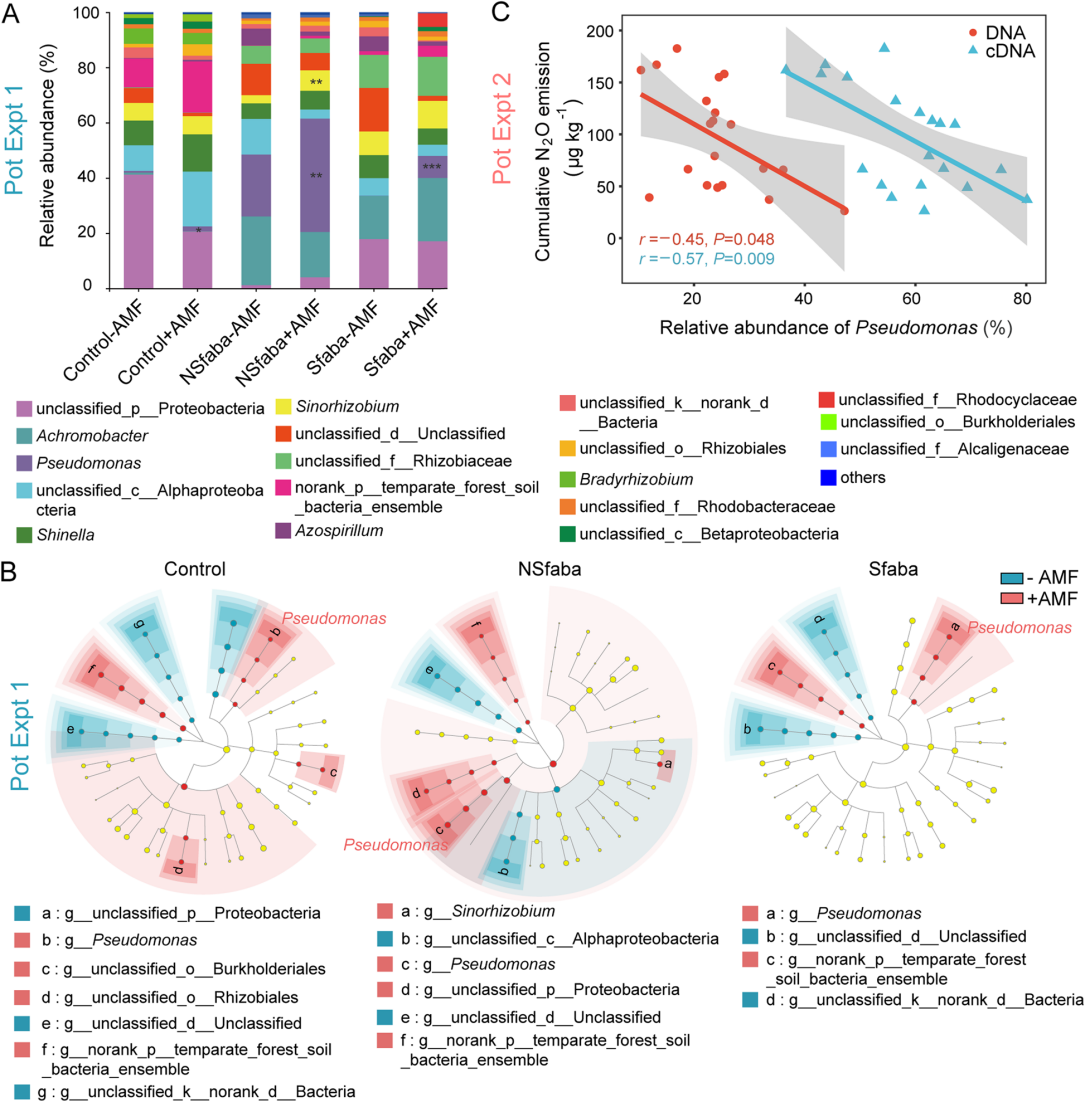
Structure of clade I *nosZ* community in three different patches in pot expt 1 (**A** and **B**), and correlation between N_2_O emissions and relative abundance of *Pseudomonas* (**C**) in pot expt 2. **A** Relative abundance of major taxonomic groups at the genus level of clade I *nosZ* community between −AMF and +AMF treatments in the control, NSfaba (unsterilized faba bean), and Sfaba (sterilized faba bean) patches (*n* = 8). Asterisks, significant differences between −AMF and +AMF treatments in each patch type according to the Wilcoxon rank sum test (*, *P* < 0.05; **, *P* < 0.01; ***, *P* < 0.001). **B** Linear discriminant analysis effect size (LEfSe) identifying the significantly different abundant taxa of clade I *nosZ* community between −AMF and +AMF treatments in the control, NSfaba, and Sfaba patches (*n* = 8). Colored shadows, trends of the significantly different taxa; only taxa meeting a linear discriminant analysis (LDA) significance threshold of > 3 are shown; Control, soil patch; NSfaba and Sfaba, patches with unsterilized or sterilized faba bean residues, respectively. **C** Correlation between cumulative N_2_O emissions and the relative abundance of *Pseudomonas* in clade I *nosZ* community. Gray shading denotes 95% confidence intervals

Amplicon sequencing analysis at the gene level in the two pot experiments and also at the transcript level in pot expt 2 was conducted to identify the N_2_O-reducing community (targeting the clade I *nosZ* community) in the residue patches. At the genus level, *Pseudomonas*, *Achromobacter*, *Shinella*, and *Sinorhizobium* were detected. *Pseudomonas* was the most abundant genus, accounting for 32% in the NSfaba patches at the gene level in pot expt 1 (Fig. 4A), and 24 and 58% at the gene and transcript levels respectively, in pot expt 2 (Fig. S3A, B). At the OTU level, AMF significantly altered the structure of the clade I *nosZ* community based on both gene (pot expts 1 and 2) and transcript (pot expt 2) analyses (Tables S4 and S5). For clade I *nosZ* community, linear discriminant analysis (LDA) effect size (LEfSe) shows that *Pseudomonas* was remarkably enriched in the presence of AMF within each patch type in pot expt 1 (Fig. 4B). Similarly, in pot expt 2, AMF significantly increased the relative abundance of *Pseudomonas* within the clade I *nosZ* community by 40% at the gene level at the first harvest (Fig. S3A) and by 27% at the transcript level at the second harvest (Fig. S3B). Moreover, cumulative N_2_O emissions were negatively correlated with the relative abundance of *Pseudomonas* at both the gene and transcript levels (*r* = −0.45, *P* < 0.05; *r* = −0.57, *P* < 0.01; Fig. 4C).

**Fig. 5:**
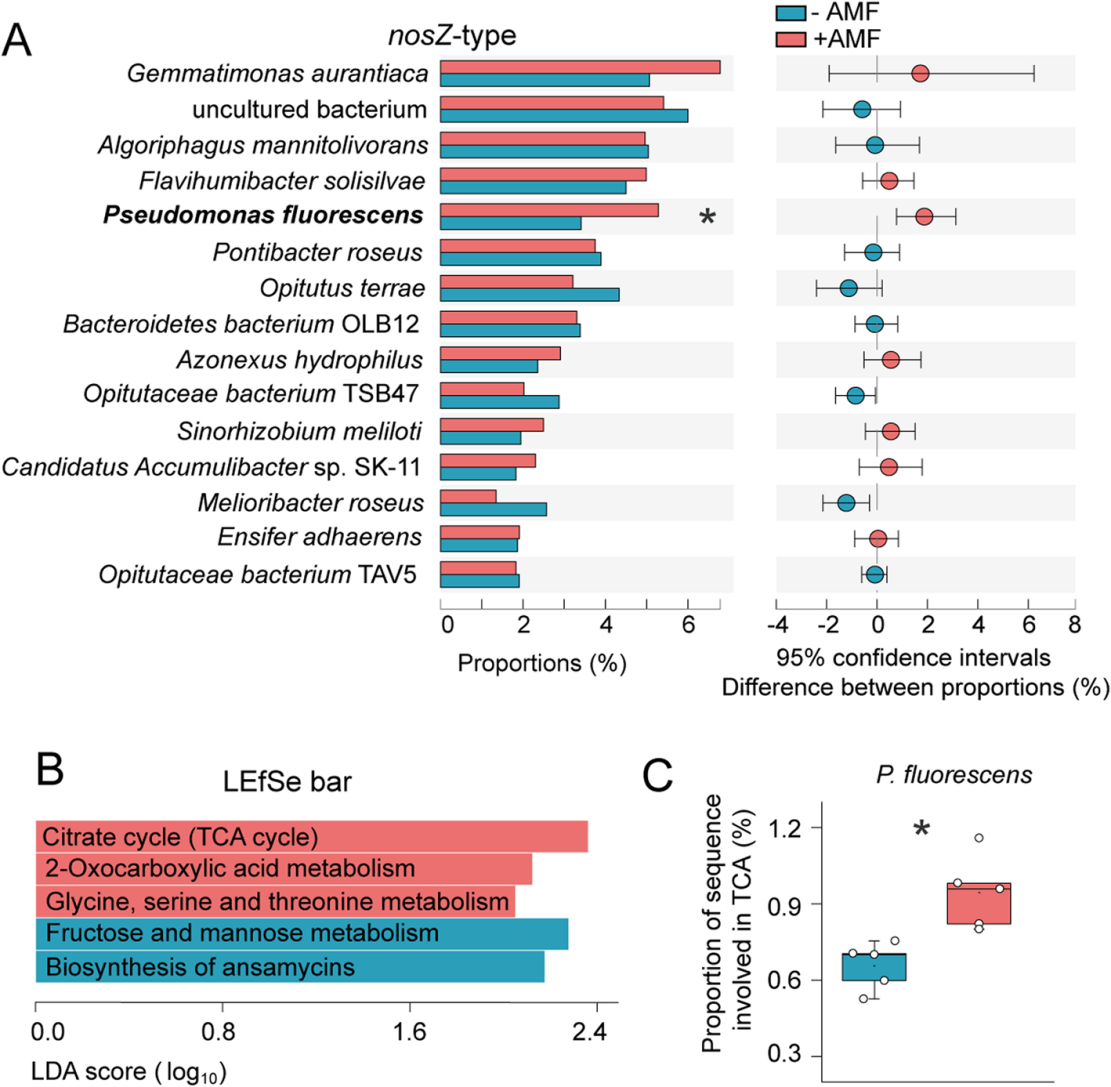
Metagenomic analysis of *Pseudomonas fluorescens* involved in the N_2_O reduction process (**A**) and carbon metabolism (**B**, **C**) at the second harvest from pot Expt 2. **A** Relative abundance of major taxonomic groups at the species level of N_2_O-reducing community between the −AMF and +AMF treatments (*n* = 5). The top 1–15 abundances are shown. **B** Linear discriminant analysis effect size (LEfSe) identifying the significantly different functions involved in carbon metabolism between the −AMF and +AMF treatments (*n* = 5). Only taxa meeting a linear discriminant analysis (LDA) significance threshold of > 2 are shown. **C** Percentages of *P. fluorescens* involved in the citrate cycle (TCA cycle) between the −AMF and +AMF treatments (*n* = 5). Asterisks, significant differences between –AMF and +AMF treatments according to the Wilcoxon rank sum test (*, *P* < 0.05). The box plots show the 25–75th percentiles (box), the median and the mean (the band and the dot inside the box), and the minimum to maximum values excluding outliers (whiskers)

Shotgun metagenomics of the microbiomes in the patches in the −AMF and +AMF treatments (pot expt 2) at the second harvest was carried out. Sequences of predicted *nosZ* genes from the KEGG database were assigned against the NCBI NR database to assess the taxonomic composition of N_2_O-reducing community. For the N_2_O-reducing community, *Pseudomonas fluorescens* was the abundant species, accounting for 4.35% on average. Only the relative abundance of *P. fluorescens* increased significantly in the +AMF treatment (Fig. 5A). The carbon metabolism and the microbial taxonomic composition were also analyzed. The relative abundances of key genes involved in the microbial citrate cycle (tricarboxylic acid [TCA] cycle) especially in *P. fluorescens*, 2-oxocarboxylic acid metabolism and glycine, serine, and threonine metabolism increased significantly in the +AMF treatment (Fig. 5B, C). Together, the altered carbon metabolism in combination with the increase in DOC content in the +AMF treatment implies that the enrichment of *P. fluorescens* and stimulation of N_2_O reductase might be associated with hyphal exudates.

### In vitro experiment: cultivation of *Pseudomonas*

A total of 40 isolates taxonomically affiliated with *Pseudomonas* were obtained from patch samples collected at the second harvest in pot expt 2. The *nosZ* gene of the 40 isolates was amplified by PCR and sequenced, with *nosZ* gene sequences detected in 27 isolates. The majority of the 27 *nosZ*-possessing isolates were aligned within the same species *Pseudomonas* JL1 and were affiliated with *P. fluorescens* based on the phylogenetic tree constructed with 16S rRNA genes (Fig. S4A). Three isolates (*P*. *fluorescens* JL1, JL2, and JL3) were then selected from the above 27 isolates to conduct draft-genome sequencing. The three isolates possessed all genes involved in complete denitrification converting nitrate into N_2_.

Using multiple sequence alignment, *nos* operon cluster analysis and the associated signal peptide (twin-arginine translocation, TAT) approaches, the selected *P. fluorescens* strain JL1 was confirmed to possess clade I TAT-dependent *nosZ* gene (100% identity to the *Pseudomonas* strain WP_047225819.1). Subsequent assays in the in vitro experiment and inoculation experiments were conducted using the *P*. *fluorescens* strain JL1. Close attachments of *P*. *fluorescens* to AMF hyphae was observed microscopically in the in vitro cultures stained with 4^′^,6-diamidino-2-phenylindole (Fig. S5A).

### In vitro experiments: chemotaxis, growth, and N_2_O production by *P. fluorescens*

**Fig. 6:**
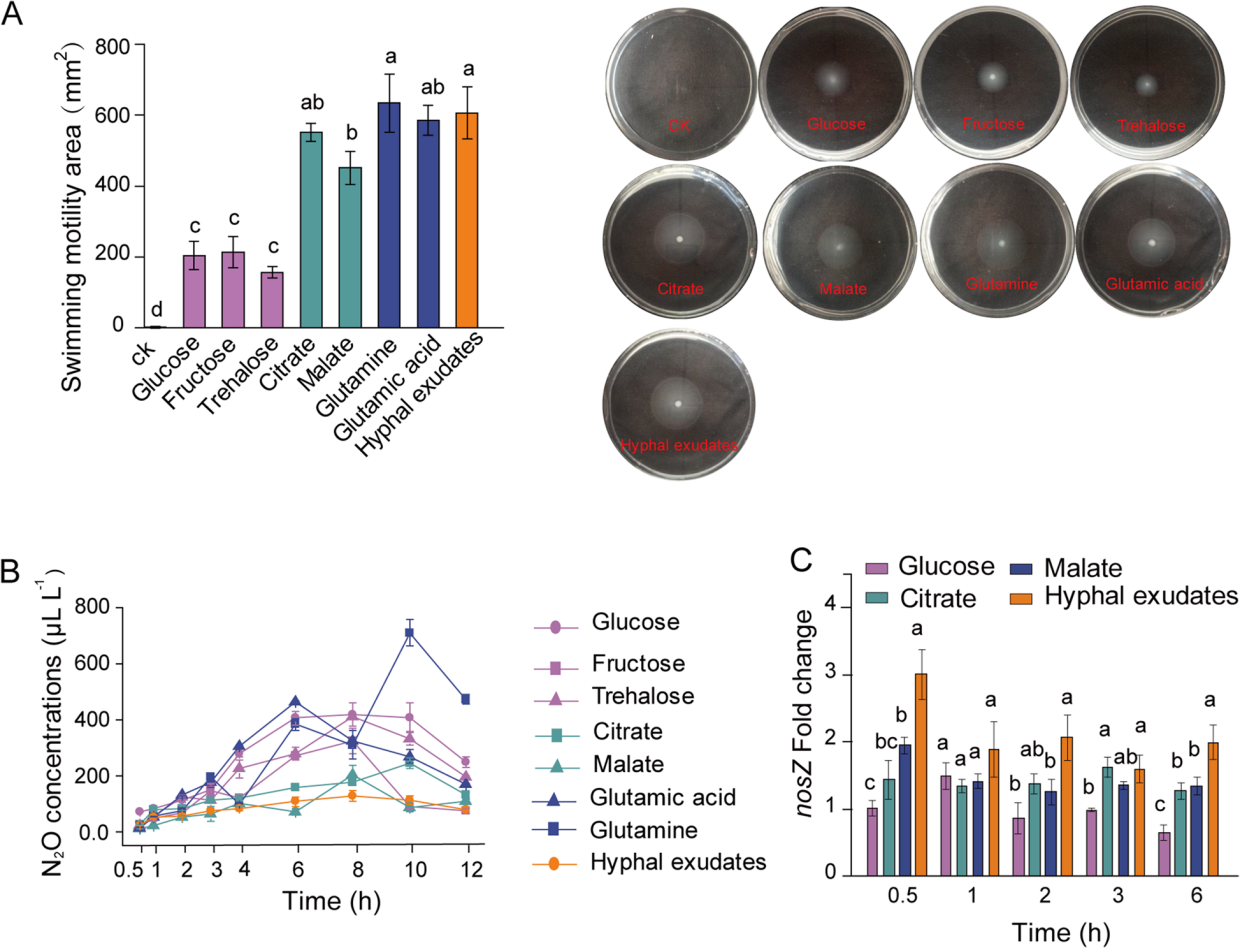
The response of *Pseudomonas fluorescens* to AMF hyphal exudates and the major compounds in the in vitro experiments. **A ***P*. *fluorescens* swimming motility area in response to AMF hyphal exudates and major compounds (*n* = 3). **B** Dynamic N_2_O concentrations in the headspace of serum bottles emitted by *P*. *fluorescens* in response to hyphal exudates and major compounds (*n* = 3). **C** Expression of the *nosZ* gene of *P*. *fluorescens* in response to hyphal exudates and major compounds (*n* = 3). Different lowercase letters indicate significant differences among treatments according to the least significant difference (LSD) test at the 5% level

Glucose, fructose, trehalose, glutamine, glutamic acid, citrate, and malate were abundant in hyphal exudates (Table S6). *P*. *fluorescens* JL1 displayed very little chemotaxis or growth in the carbon-free medium but its chemotaxis and growth increased quickly upon the addition of hyphal exudates (Fig. 6A and Fig. S5B). The areas of swimming motility (indicating chemotactic ability) of *P*. *fluorescens* JL1 in the media supplemented with amino acids (glutamine and glutamic acid) or carboxylates (citrate and malate) were comparable to those obtained with hyphal exudates, which were on average three times higher than those obtained with sugars (glucose, fructose, and trehalose) (Fig. 6A). However, the optical densities (ODs) of *P*. *fluorescens* JL1 in the media supplemented with amino acids and citrate were higher than those obtained with hyphal exudates and sugars (Fig. S5B).

*P. fluorescens* JL1 was cultured anaerobically to study the effects of hyphal exudates and major compounds on N_2_O emission and the expression of *nirS* and *nosZ* genes. Indeed, the N_2_O concentrations in *P. fluorescens* JL1 cultures receiving hyphal exudates or carboxylates (citrate, malate) were significantly lower than those in cultures receiving amino acids or sugars over the incubation period (Fig. 6B). Furthermore, the *nosZ* gene expression and the transcript ratio of *nosZ*/*nirS* (except at 1 h) were highest in cultures receiving hyphal exudates, followed by the citrate or malate addition treatments, and the values in the glucose addition treatment was the lowest (Fig. 6C and Fig. S5C).

### Inoculation experiment: validation that AMF exudates stimulated nosZ gene expression and reduced N_2_O production by *P. fluorescens*

**Fig. 7:**
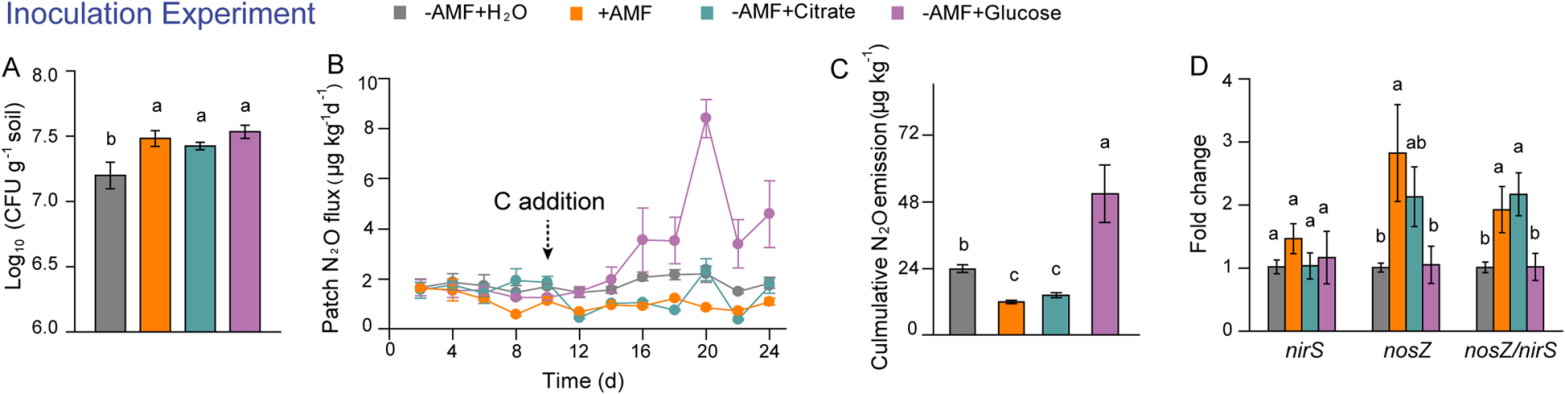
Response of *Pseudomonas fluorescens* to AMF hyphal exudates and the major compounds in the inoculation experiment. **A** Numbers (CFU) of *P*. *fluorescens* in patches in response to AMF, glucose, and citrate (*n* = 4). **B**, **C** N_2_O flux (B) and cumulative N_2_O emission (C) from patches inoculated with *P*. *fluorescens* in response to AMF, glucose, and citrate (*n* = 4). **D** Expression of the *nosZ* gene in patches inoculated with *P*. *fluorescens* in response to AMF, glucose, and citrate (*n* = 4). Different lowercase letters indicate significant differences among treatments by the least significant difference (LSD) test at the 5% level following one-way analysis of variance (*P* < 0.05)

An experiment with the re-inoculation of sterilized residue patches with *P*. *fluorescens* strain JL1 was conducted to determine how AMF colonization and/or AMF exudates stimulated *nosZ* gene expression and hampered N_2_O production (Fig. 1). Here, the bacterial numbers were > 10^7^ CFU g^−1^ soil in patches inoculated with *P*. *fluorescens* JL1. The bacterial numbers in the +AMF and −AMF + citrate/glucose treatments were significantly higher than those in the −AMF+H_2_O treatments (Fig. 7A). Twelve days after patch addition and 2 days after carbon addition, the N_2_O fluxes were significantly lower in the +AMF and −AMF + citrate treatments than in the −AMF and −AMF + glucose treatments (Fig. 7B). Cumulative N_2_O emissions in the +AMF and −AMF + citrate treatments were 50 and 40% lower, respectively, than in the −AMF + H_2_O treatment, and approximately 80% lower than in the −AMF + glucose treatment (Fig. 7C). Compared to the −AMF + H_2_O/glucose treatments, *nosZ* gene expression was upregulated in the +AMF treatment and the transcript ratio of *nosZ*/*nirS* increased in the +AMF and −AMF + citrate treatments (Fig. 7D).

### Field experiment: correlation between AMF and the abundance of clade I *nosZ* gene

**Fig. 8:**
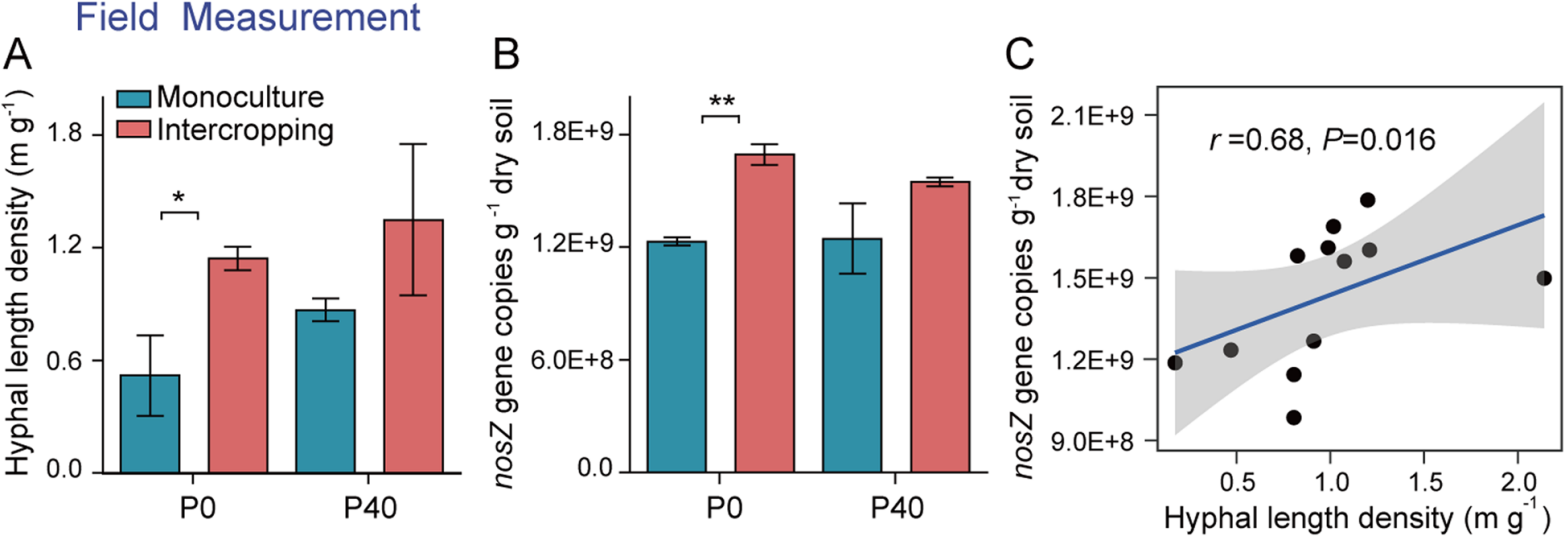
Hyphal length density, *nosZ* gene copies, and their correlation in the long-term field experiment. **A**, **B** Hyphal length density (**A**) and *nosZ*-type gene copies (**B**) in long-term field soils of faba bean monoculture and maize/faba bean intercropping under different phosphate fertilizer application rates (*n* = 3). Asterisks, significant differences between monoculture and intercropping within each P application rate according to the two-tailed unpaired *t* test (*, *P* < 0.05; **, *P* < 0.01). **C** Correlation between hyphal length density and *nosZ* gene copies. Correlation analysis is based on the Spearman correlation coefficient. Gray shading denotes 95% confidence intervals

We took samples from an 11-year-long intercropping field experiment. HLD and the abundance of clade I *nosZ* gene in the maize/faba bean intercropping treatment were significantly higher than in the faba bean monoculture under zero P application (Fig. 8A, B). Furthermore, the abundance of clade I *nosZ* gene was significantly positively correlated with HLD (Fig. 8C).

## Discussion

Returning crop residues to the field is an effective measure to increase carbon sequestration in agricultural ecosystems but this gain can be offset by high N_2_O emission, especially when residues of N_2_-fixing legumes are returned [40]. Crop residues in soils create unique micro-environmental conditions that are conducive to denitrification by absorbing water from surrounding soil and by stimulating microbial respiration due to dissolved organic carbon released during decomposition [2, 5]. The current study clearly demonstrates that (i) interactions between AMF and N_2_O reducers mitigate N_2_O emissions in residue patches, as evidenced by the alteration in N_2_O flux and the changes in the abundance and community composition of hyphosphere microbiota in the two pot experiments; and (ii) carboxylates exuded by hyphae recruited complete denitrifier (*P*. *fluorescens*) and triggered the *nosZ* gene (encoding N_2_O reductase) expression of *P. fluorescens*, as evidenced by the chemotaxis, growth, and N_2_O production in the in vitro cultures and the inoculation experiment.

### Interactions between AMF and N_2_O reducers mitigate N_2_O emission in patches

In pot expt 1, the presence of AMF hyphae suppressed N_2_O concentrations in the unsterilized faba bean (NSfaba) patches after NO_3_^−^ application but not after NH_4_^+^ application (Fig. 2A and Fig. S1B). In pot expt 2, the size of the patches was enlarged to 202 g and NO_3_^−^ was supplied as basal fertilizer to all chambers including patch chambers to minimize N diffusion. The residue rate (10 g kg^−1^) was comparable to crop residues used in previous studies under field and condition-controlled conditions [41–43]. Here again AMF hyphae consistently and significantly reduced the N_2_O flux from residue patches from day 8 after patch placement until the end of the experiment (Fig. 2B). The consistent results in the two experiments provide compelling evidence that AMF hyphae reduced N_2_O emissions in the residue patches, primarily by mediating the denitrification pathway, although the relative importance of this pathway among other processes may merit further exploration [6]. Our results are in line with previous studies showing AMF-mediated reduction of N_2_O emission from soil with residue amendment [20, 21] or without residue amendment under high soil moisture conducive to denitrification [23, 24, 44].

The diversity and activity of the N_2_O-producing (*nirK* or *nirS* type) and N_2_O-reducing (*nosZ* type) microbial communities ultimately determine net N_2_O emissions. The relative abundance of bacteria possessing the *nosZ* gene is a good proxy of the N_2_O/ (N_2_ + N_2_O) ratio [45]. In pot expt 1, AMF hyphae significantly increased the abundance of clade I *nosZ,* the *nirS* gene, and the *nosZ* I/(*nirK + nirS*) ratio in the NSfaba patches (Fig. 3A). As there was higher frequency of co-occurrence of *nosZ* with *nirS* [9], these results indicate that AMF hyphae may promote the growth and expression of N_2_O reducers (clade I) in residue patches. This was further supported by pot expt 2 where AMF significantly increased the abundance and expression of clade I *nosZ*, and the transcript ratio of *nosZ* I/(*nirK*+*nirS*) at the second but not at the first harvest (Fig. 3B, C). Synergies between AMF and N_2_O reducers may therefore explain the decline in N_2_O production in residue patches. In pot expt 2 at the first harvest, the increase in the expression of the *nirK* gene (Fig. 3C) might be a response to imposing anaerobiosis which primes an initial pulse of emission. Hence, research efforts on dynamic changes of N_2_O reducer/producer community are required in future. In our experiment, no significant difference in the abundance and expression of clade II *nosZ* was observed between the −AMF and +AMF treatments (Fig. 3B, C), suggesting these bacteria may be of relatively minor importance compared to clade I type. Previous studies showed that the clade I *nosZ* was dominant in the rhizosphere while clade II was in the soils [46]. It is likely that in similar fashion to (mycor-)rhizosphere, hyphosphere generated by the proliferation of AMF into the residues is favorable for the clade I *nosZ* community.

The N rate applied to the patches (approximately 200 mg kg^−1^) was equivalent to the amount of fertilizer N typically used for cereal crops [47, 48]. High concentrations of NO_3_^−^ in soil almost completely inhibit N_2_O reduction to N_2_ [49], as NO_3_^−^ reductase outcompetes N_2_O reductase for electrons [50] supplied by labile organic carbon including AMF exudates. A recent study shows that the reduction in the rate of N_2_O emissions in the presence of AMF under normal N inputs was higher than that under high N inputs in conventional soil, but the opposite trend occurred in organically managed soil [44]. Aside from the well-reported substrate-controlled denitrification process [49], the interactions of AMF and hyphospheric microbes are also shown to be regulated by nitrogen availability [22]. Yet this remains largely unexplored. It is therefore particularly desirable to investigate AMF-mediated denitrification mechanisms in the context of environmental controls in order to maximize the N_2_O mitigation potential of AMF.

### Exudation of carboxylates by AMF hyphae recruits *P. fluorescens* and triggers *nosZ* gene expression in *P. fluorescens*

Soils contain diverse denitrifying bacteria such as *Citrobacter*, *Pseudomonas*, *Ochrobactrum*, and *Burkholderia* [51, 52]. A previous study reported that only a few members of the bacterial community (~10%) in residue patches responded to AMF colonization according to 16S rRNA gene microarray analysis [53]. The results obtained from amplicon and metagenomic sequencings in pot experiments and isolation in the in vitro cultures supported the conclusion that AMF hyphae consistently increased the relative abundance of N_2_O-reducing *Pseudomonas*, which was predominant in residue patches (Figs. 4A and 5A and Fig. S3). Moreover, cumulative N_2_O emissions were negatively correlated with the relative abundance and activity of *Pseudomonas* (Fig. 4C). This is the first report of N_2_O-reducing *Pseudomonas* directly and positively responded to AMF hyphal proliferation being responsible for low N_2_O emissions in residue patches. *Pseudomonas* spp. are fast-growing *r*-strategists enriched in nutrient-rich environments such as the rhizosphere [54] and hyphosphere [55]. In a similar fashion to the rhizosphere, the hyphosphere provides a unique niche in which microbial communities differ from those in the bulk soil due to hyphal exudates [53, 56], as supported by the increased patch DOC concentrations in the +AMF treatment (Fig. S1C). Most *Pseudomonas* isolates cultivated in vitro possessing the *nosZ* gene belonged to *P. fluorescens* (Fig. S4A). The three isolates (*P*. *fluorescens* JL1, JL2, and JL3) selected for draft-genome sequencing possessed all denitrifying genes and were complete denitrifiers. *P. fluorescens* F113 was previously reported as a typical “true denitrifier” [12]. *P. fluorescens* is effectively attached to AMF hyphae (Fig. S5A), as was also observed in a previous study [57]. Taken together, these results imply that the enrichment and stimulation of complete denitrifying *P. fluorescens* in the hyphosphere can be attributed to AMF hyphal exudates.

AMF hyphae exude organic carbon, mainly in the form of sugars, carboxylates, and amino acids [58, 59]. Previous studies show that AMF hyphal exudates promoted the growth of phosphate-solubilizing bacteria and that fructose exuded by AMF stimulated the expression of phosphatase genes in *Rahnella aquatilis* [16, 17]. Here, we found that glucose, fructose, trehalose, glutamine, glutamic acid, citrate, and malate were abundant in hyphal exudates (Table S6), corroborating with previous studies [59, 60]. AMF hyphal exudates significantly promoted the chemotaxis and growth of *P. fluorescens* (Fig. 6A and Fig. S5B), reduced N_2_O emissions, and upregulated the expression of the *nosZ* but not of the *nirS* gene (Fig. 6B, C and Fig. S5C). Moreover, the role of carboxylates in bacterial chemotaxis, N_2_O emissions, and gene expression was similar to that of hyphal exudates (Fig. 6). Together, these results demonstrate that carboxylates exuded by hyphae are attractants in recruiting *P*. *fluorescens* and also act as stimulants triggering *nosZ* gene expression, resulting in a significant decline in N_2_O emissions. This was further validated in the inoculation experiment in which cumulative N_2_O emission and *nosZ* gene expression in the citrate addition treatment were similar to those in the +AMF treatment and in which N_2_O emission lower and *nosZ* gene expression higher than in the glucose or H_2_O addition treatments (Fig. 7C, D). Thus, an N_2_O-reducing microbiome in residue patches has been developed by carboxylates exuded by AMF hyphae. A similar situation with reduced N_2_O emissions after the addition of carboxylates such as citrate, succinate, and acetate but not glucose to soils [61] or to pure cultures of *Pseudomonas* [62] was previously observed.

The N_2_O reductase encoded by the *nosZ* gene is a weak competitor for electrons compared to other denitrifying reductases [50]. NADH, the usual direct electron donor mainly produced in the citrate cycle, is more conducive to electron transfer to N_2_O reductase. Carboxylates such as citrate and malate in hyphal exudates are directly involved in the citrate cycle, while the metabolic use of glucose requires enzymatic conversions and consumes extra energy [63, 64]. Moreover, AMF increased the relative abundances of key genes involved in citrate cycle of bacteria, especially *P. fluorescens* in residue patches (pot expt 2, Fig. 5B, C). Taken together, these results imply that hyphal exudates (with carboxylates as major components) promote the citrate cycle, trigger complete denitrification, and subsequently reduce N_2_O emissions by *P*. *fluorescens*.

The results of the current study may be relevant for diverse ecosystems. The values of HLD in the present study fall within the range of 200–600 cm cm^−3^ (approximately 1.5–5.0 m g^−1^) in farmland soil but were lower than in woody and non-woody systems (2400 and 2700 cm cm^−3^ on average, respectively) [14]. The global decline in the abundance and diversity of AMF due to increasing land use intensity [65] is potentially alarming. This decline may disrupt the extensive connections between AMF and their associated microbiomes, with cascading negative effects on ecosystem functioning, specifically with respect to the underappreciated role of co-colonization by AMF and *Pseudomonas* in the mitigation of N_2_O emissions. To counter this adverse development, the restoration of AMF diversity in agricultural ecosystems may be achieved by the development of sustainable management practices such as diversified cropping [66], organic farming [67], or conservation agriculture [68]. To verify that sustainable agriculture practices may indeed stimulate co-colonization by AMF and N_2_O reducers, we analyzed soil samples taken from an 11-year-long intercropping field experiment. In the maize/faba bean intercropping soils, the HLD and the gene abundance of clade I *nosZ* were significantly higher than those in the faba bean monoculture, and the clade I *nosZ* gene abundance was significantly positively correlated with HLD (Fig. 8C). Similar situations, i.e., low mineral N and high organic C availability, may also occur in grassland and forest soils, where uptake of atmospheric N_2_O is observed [69]. We speculate that the mechanisms we describe in the present study may explain this phenomenon, as AMF are abundant in these ecosystems. Our study demonstrates that reinforcing synergies between AMF and the hyphosphere microbiome may have far-reaching implications for both sustainable agriculture and the mitigation of N_2_O emissions from cropping systems and, thus, for the mitigation of climate change. We envisage that indiscernible and variable N_2_O fluxes occurring in soil microenvironments can be substantially reduced by AMF and the hyphosphere microbiome. Our study therefore also advances our understanding of the multiple functions delivery by AMF beyond promoting uptake of soil nutrients.

## Conclusions

Our study provides novel insights into the importance of AMF in mediating nitrogen transformation processes conducted mainly by denitrifiers that lead to cascading effects on soil N_2_O emission. We demonstrate that AMF enriched the N_2_O-reducing *Pseudomonas* in the hyphosphere, which was responsible for the decline in N_2_O emissions in the residue patches. Notably, carboxylates exuded by hyphae acted as attractants recruiting *P*. *fluorescens* JL1 and as stimulants triggering the expression of *nosZ* gene. These insights provide a novel mechanistic understanding of the intriguing interactions between AMF and microbial guilds in the hyphosphere, and collectively indicate how these trophic microbial interactions substantially affect the denitrification process at microsites. This knowledge opens novel avenues to exploit cross-kingdom microbial interactions for sustainable agriculture and climate change mitigation.

## Supplementary Information

**Additional file 1: Fig. S1.** Hyphal length density (pot expt 1), patch N_2_O concentrations (pot expt 1) dissolved organic carbon content, and total carbon and nitrogen contents (pot expt 2) in patches in the absence or presence of AMF. A, pot expt 1. Hyphal length density from different patches under −AMF and +AMF treatments (*n* = 8). B, pot expt1. Dynamic N_2_O concentrations from different patches under −AMF and +AMF treatments after the addition of NO_3_^−^-N or NH_4_^+^-N (*n* = 4). Control, soil patch; NSfaba and Sfaba; patches with unsterilized (NS) or sterilized (S) faba bean residues, respectively. C-F, pot expt 2. Dissolved organic carbon (C), hyphal length density (D), total carbon (E) and total nitrogen (F) content under the −AMF and +AMF treatments at both harvests (*n* = 5). T1 and T2, the first (day 24) and second (day 34) harvests, respectively; asterisks, significant differences between the −AMF and +AMF treatments in each patch type (pot expt 1) or at each harvest (pot expt 2) according to two-tailed unpaired *t*-tests (*, *P* < 0.05; **, *P* < 0.01; ***, *P* < 0.001).

**Additional file 2: Fig. S2.** Correlation between N_2_O emission, hyphal length density and *nosZ* gene copies or transcript copies. A, pot expt 1. Correlation between N_2_O concentration and *nosZ* gene copies in different patch types 24 h after the addition of ammonium or nitrate. B, pot expt 1. Correlation between *nosZ* gene copies and hyphal length density in different patche types. Control, soil patch; NSfaba and Sfaba, patches with unsterilized (NS) or sterilized (S) faba bean residues, respectively. C, D, pot expt 2. Correlation of *nosZ* gene and transcript copies with hyphal length density (C) and dissolved organic carbon (D) contents at the first and second harvests. Correlation analysis is based on Pearson correlation coefficient. Gray shading denotes the 95% confidence intervals, and only significant correlations are listed.

**Additional file 3: Fig. S3.** Structure of microbial communities harbouring *nirK*, *nirS* and clade I *nosZ* in pot expt 2. A, B, The relative abundance of major taxonomic groups of *nirK*, *nirS* and clade I *nosZ* communities in the absence or presence of AMF at both harvests based on gene (A) and transcript (B) levels (*n* = 5). T1 and T2, the first (day 24) and second (day 34) harvests, respectively; asterisks, significant differences between the −AMF and +AMF treatments at each harvest according to the Wilcoxon rank sum test (*, *P* < 0.05; **, *P* < 0.01; ***, *P* < 0.001).

**Additional file 4: Fig. S4.** Phylogeny and community structure of culturable denitrifying bacteria in response to AMF hyphae in the in vitro experiment. A, Phylogenetic tree of culturable denitrifying bacteria from patches of faba bean residue. This was constructed by the neighbor-joining method based on 16S rRNA gene sequences. Names of strains obtained from this study are shown in bold. B, Relative abundances of major culturable denitrifying bacterial communities in the absence or presence of AMF (*n* = 5). Asterisks, significant differences between the −AMF and +AMF treatments according to the Wilcoxon rank sum test (*, *P* < 0.05; **, *P* < 0.01; ***, *P* < 0.001). C, Nonmetric multidimensional scaling (NMDS) pattern of culturable denitrifying bacterial communities between −AMF and +AMF treatments based on Bray–Curtis dissimilarity. Ellipses in the plots indicate 95% confidence intervals for microbial communities under the −AMF and +AMF treatments (*n* = 5).

**Additional file 5: Fig. S5.** Response of *Pseudomonas fluorescens* to AMF hyphal exudates and major compounds in the in vitro experiment. A, AMF hyphae with attached *P. fluorescens* stained with 4′,6-diamidino-2-phenylindole (DAPI); scale bar, 10 μm. B, Bacterial optical densities (OD_600_) of *P*. *fluorescens* in response to AMF hyphal exudates and major compounds (*n* = 3). C, Expression of the *nirS* gene and *nosZ*/*nirS* ratio of *P*. *fluorescens* in response to hyphal exudates and major compounds (*n* = 3). Different lowercase letters indicate significant differences among treatments by the least significant difference (LSD) test at the 5% level. D, Dynamic N_2_O concentrations in the headspace of serum bottles emitted from three strains of *P*. *fluorescens* in response to glucose, citrate, and hyphal exudates (*n* = 3). Asterisks, significant differences between hyphal exudate or citrate treatment and glucose treatment at 3 h within each strain according to two-tailed unpaired *t*-test (*, *P *< 0.05; ***, *P* < 0.001) .

**Additional file 6: Fig. S6.** Soil water content, total carbon and nitrogen contents, mineral nitrogen and dissolved total nitrogen contents in patches in the absence or presence of AMF A-C, pot expt 1. Soil water content (A), total carbon (B) and nitrogen (C) contents under the −AMF and +AMF treatments in different patches (*n* = 8). Control, soil patch; NSfaba and Sfaba, patches with unsterilized (NS) or sterilized (S) faba bean residues, respectively. D-G, pot expt 2. Soil water content (D), ammonium (E), nitrate (F) and dissolved total nitrogen (G) contents under the −AMF and +AMF treatments at both harvests (*n* = 5). T1 and T2, the first (day 24) and second (day 34) harvests, respectively; Asterisks, significant differences between −AMF and +AMF treatments at each harvest (pot Expt 2) according to two-tailed unpaired *t*-test (*, *P* < 0.05; **, *P* < 0.01; ***, *P* < 0.001).

**Additional file 7: Fig. S7.** Structure of *nirK* and *nirS* communities in the absence or presence of AMF. Pot expt 1. Relative abundance of major taxonomic groups of *nirK* and *nirS* communities under the −AMF and +AMF treatments in different patches (*n* = 8). Control, soil patch; NSfaba and Sfaba, patches with unsterilized (NS) or sterilized (S) faba bean residues, respectively; asterisks, significant differences between the −AMF and +AMF treatments in each patch type according to the Wilcoxon rank sum test (*, *P* < 0.05; **, *P* < 0.01; ***, *P* < 0.001).

**Additional file 8: **Materials and Methods. Supplementary Text. **Table S1.** Temporal N_2_O concentrations (μL L^-1^) in the headspace in the preliminary experiment. **Table S2.** Primers and PCR conditions used for the PCR. **Table S3.** Stepwise multiple regression to identify the abundance and expression of key genes involved in N cycling which had the strongest statistical contributions to variation in the cumulative N_2_O emission in pot expt 2. Independent variables include the abundances and expressions of *nirK*, *nirS* and clade I and II *nosZ* genes. Dependents variable is the cumulative N_2_O emission. **Table S4.** Permutational multivariate analysis of variance (PERMANOVA) of the effects of patch type (PT; pot expt 1) or harvest time (HT; pot expt 2) and AMF treatment on microbial communities harbouring *nirK*, *nirS* and clade I *nosZ* based on the gene and transcript sequencing. **Table S5.** Permutational multivariate analysis of variance (PERMANOVA) of the effect of AMF treatment on clade I *nosZ* community in different patches (pot expt 1) or harvest time (pot expt 2) based on the gene and transcript sequencing. **Table S6.** In vitro experiment: metabolite concentrations in the hyphal exudates of *Rhizophagus irregularis*. **Table S7.** Effects of patch type and AMF treatment on biomass, N concentration and N content of maize in pot expt 1. **Table S8.** Effects of harvest time and AMF treatment on biomass, N concentration and N content of maize in pot expt 2.

## Data Availability

Supplementary Information contains additional data and results. The sequences have been submitted to the NCBI database (PRJNA804317).
