# Supplementary figures and images for: Mycorrhiza-mediated recruitment of complete denitrifying Pseudomonas reduces N2O emissions from soil
Source: Microbiome. 2023 Mar 9;11:45. doi: 10.1186/s40168-023-01466-5 (PMC9996866; doi:10.1186/s40168-023-01466-5)

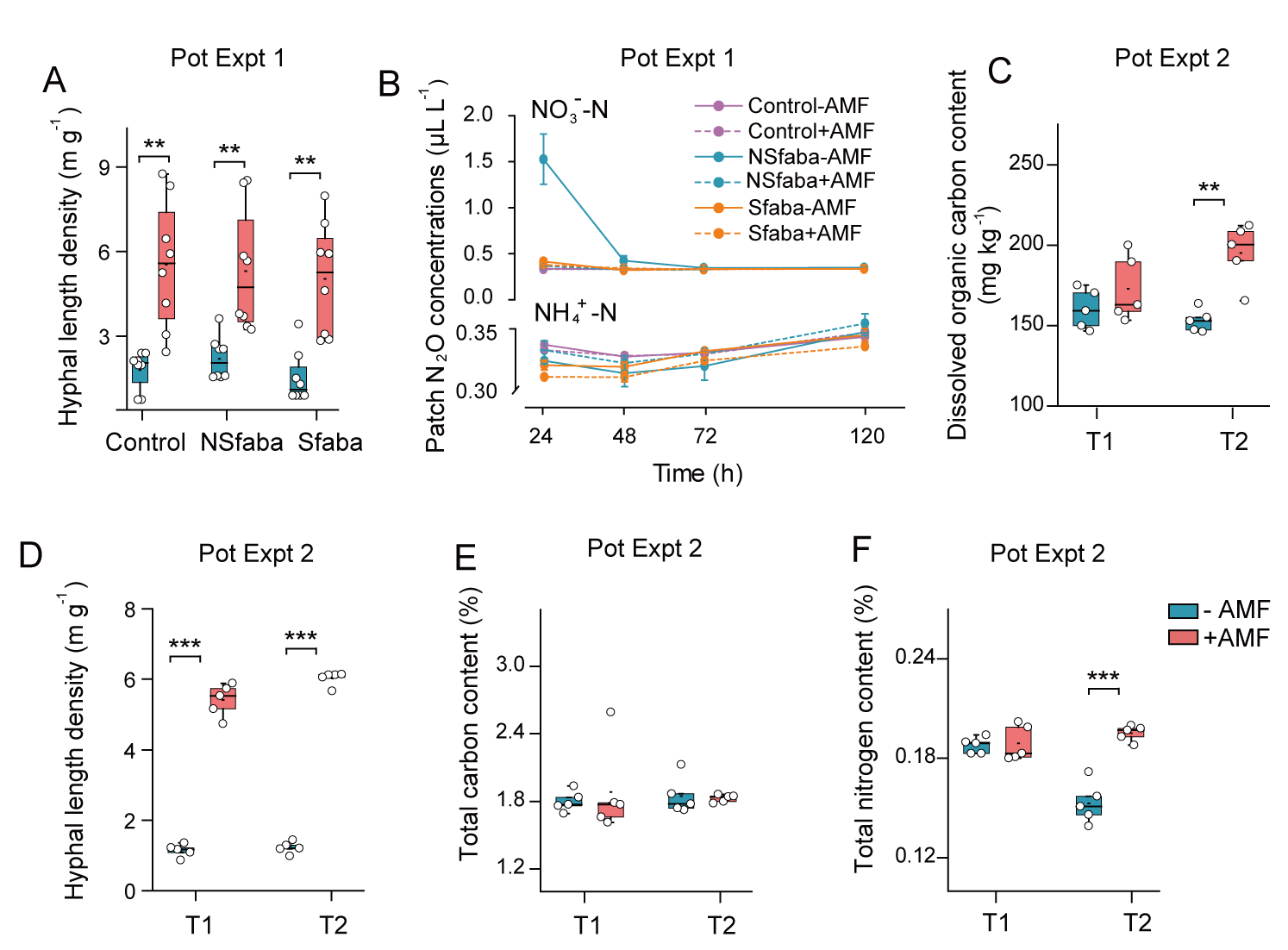

Supplement: Supplementary file 2 — Additional file 1: Fig. S1. Hyphal length density (pot expt 1), patch N2O concentrations (pot expt 1) dissolved organic carbon content, and total carbon and nitrogen contents (pot expt 2) in patches in the absence or presence of AMF. A, pot expt 1. Hyphal length density from different patches under −AMF and +AMF treatments (n = 8). B, pot expt1. Dynamic N2O concentrations from different patches under −AMF and +AMF treatments after the addition of NO3−-N or NH4+-N (n = 4). Control, soil patch; NSfaba and Sfaba; patches with unsterilized (NS) or sterilized (S) faba bean residues, respectively. C-F, pot expt 2. Dissolved organic carbon (C), hyphal length density (D), total carbon (E) and total nitrogen (F) content under the −AMF and +AMF treatments at both harvests (n = 5). T1 and T2, the first (day 24) and second (day 34) harvests, respectively; asterisks, significant differences between the −AMF and +AMF treatments in each patch type (pot expt 1) or at each harvest (pot expt 2) according to two-tailed unpaired t-tests (*, P < 0.05; **, P < 0.01; ***, P < 0.001). [file 40168_2023_1466_MOESM1_ESM.tif]

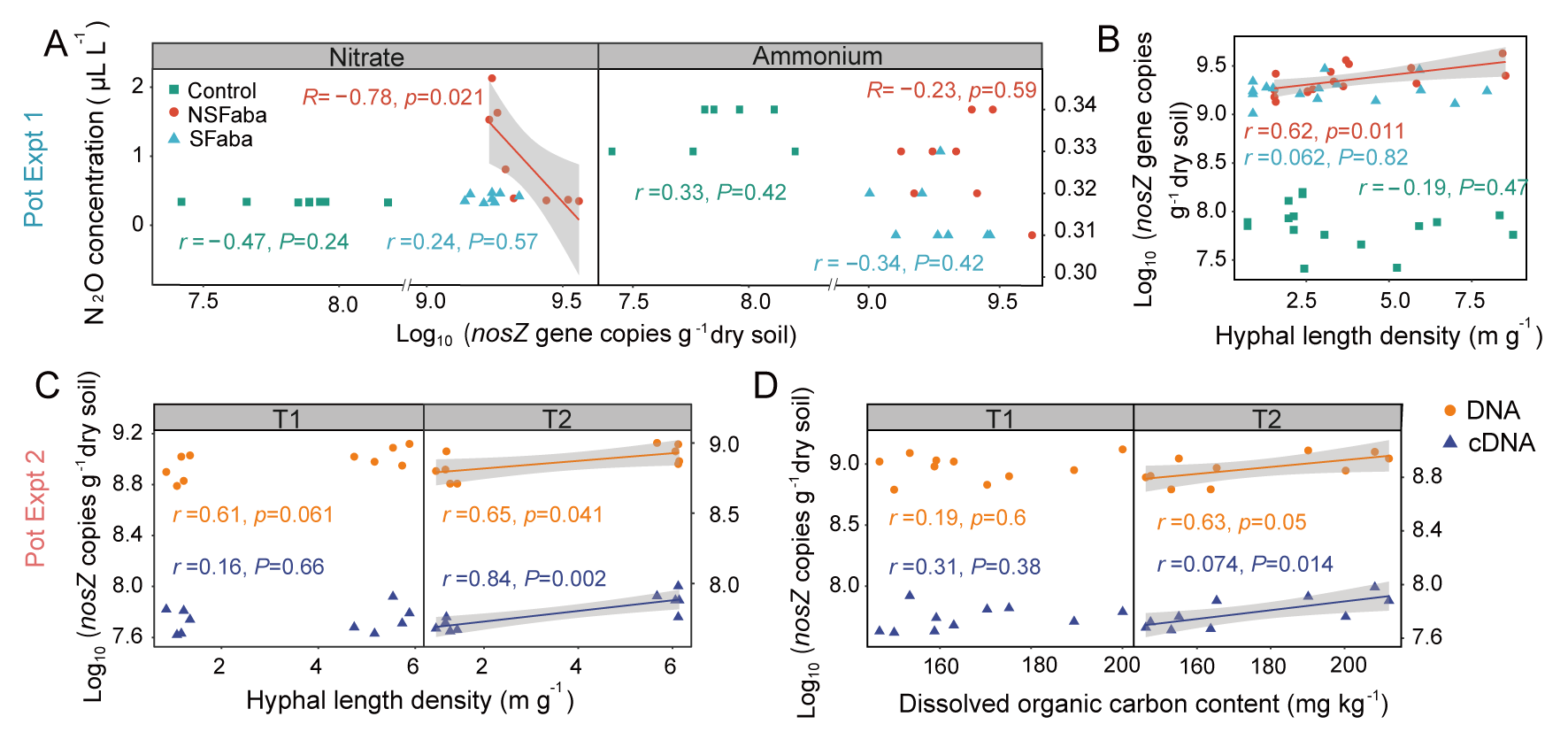

Supplement: Supplementary file 3 — Additional file 2: Fig. S2. Correlation between N2O emission, hyphal length density and nosZ gene copies or transcript copies. A, pot expt 1. Correlation between N2O concentration and nosZ gene copies in different patch types 24 h after the addition of ammonium or nitrate. B, pot expt 1. Correlation between nosZ gene copies and hyphal length density in different patche types. Control, soil patch; NSfaba and Sfaba, patches with unsterilized (NS) or sterilized (S) faba bean residues, respectively. C, D, pot expt 2. Correlation of nosZ gene and transcript copies with hyphal length density (C) and dissolved organic carbon (D) contents at the first and second harvests. Correlation analysis is based on Pearson correlation coefficient. Gray shading denotes the 95% confidence intervals, and only significant correlations are listed. [file 40168_2023_1466_MOESM2_ESM.tif]

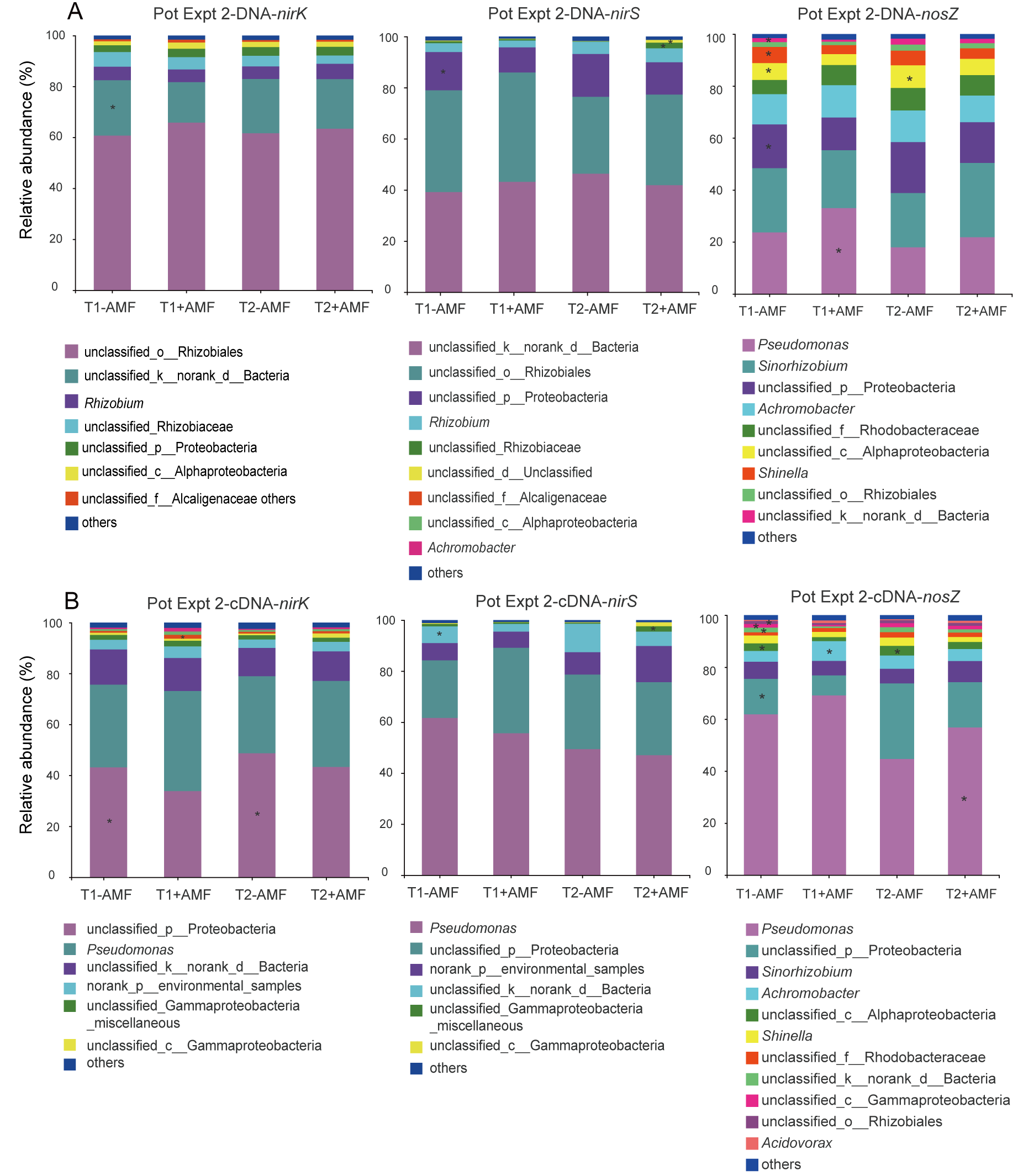

Supplement: Supplementary file 4 — Additional file 3: Fig. S3. Structure of microbial communities harbouring nirK, nirS and clade I nosZ in pot expt 2. A, B, The relative abundance of major taxonomic groups of nirK, nirS and clade I nosZ communities in the absence or presence of AMF at both harvests based on gene (A) and transcript (B) levels (n = 5). T1 and T2, the first (day 24) and second (day 34) harvests, respectively; asterisks, significant differences between the −AMF and +AMF treatments at each harvest according to the Wilcoxon rank sum test (*, P < 0.05; **, P < 0.01; ***, P < 0.001). [file 40168_2023_1466_MOESM3_ESM.tif]

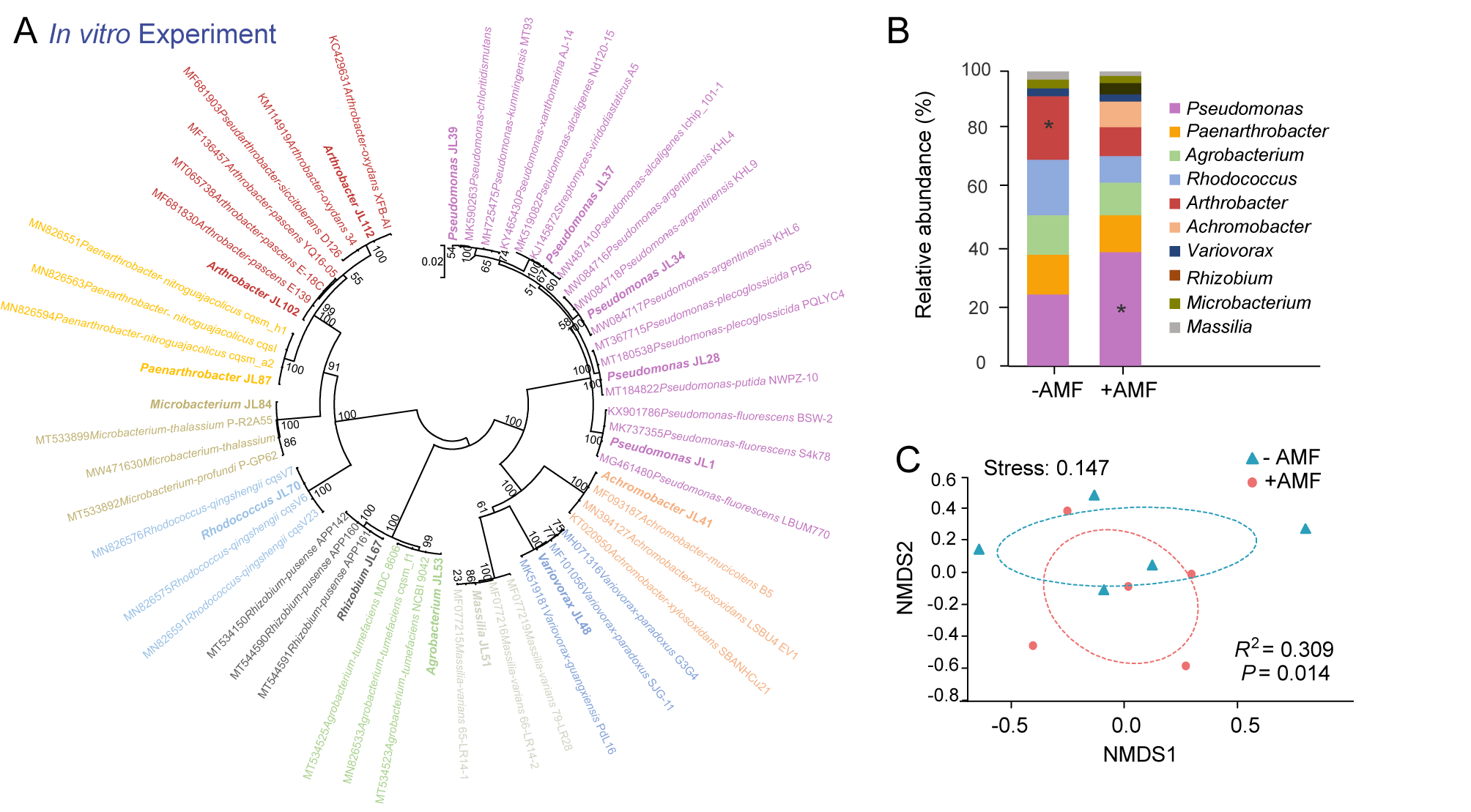

Supplement: Supplementary file 5 — Additional file 4: Fig. S4. Phylogeny and community structure of culturable denitrifying bacteria in response to AMF hyphae in the in vitro experiment. A, Phylogenetic tree of culturable denitrifying bacteria from patches of faba bean residue. This was constructed by the neighbor-joining method based on 16S rRNA gene sequences. Names of strains obtained from this study are shown in bold. B, Relative abundances of major culturable denitrifying bacterial communities in the absence or presence of AMF (n = 5). Asterisks, significant differences between the −AMF and +AMF treatments according to the Wilcoxon rank sum test (*, P < 0.05; **, P < 0.01; ***, P < 0.001). C, Nonmetric multidimensional scaling (NMDS) pattern of culturable denitrifying bacterial communities between −AMF and +AMF treatments based on Bray–Curtis dissimilarity. Ellipses in the plots indicate 95% confidence intervals for microbial communities under the −AMF and +AMF treatments (n = 5). [file 40168_2023_1466_MOESM4_ESM.tif]

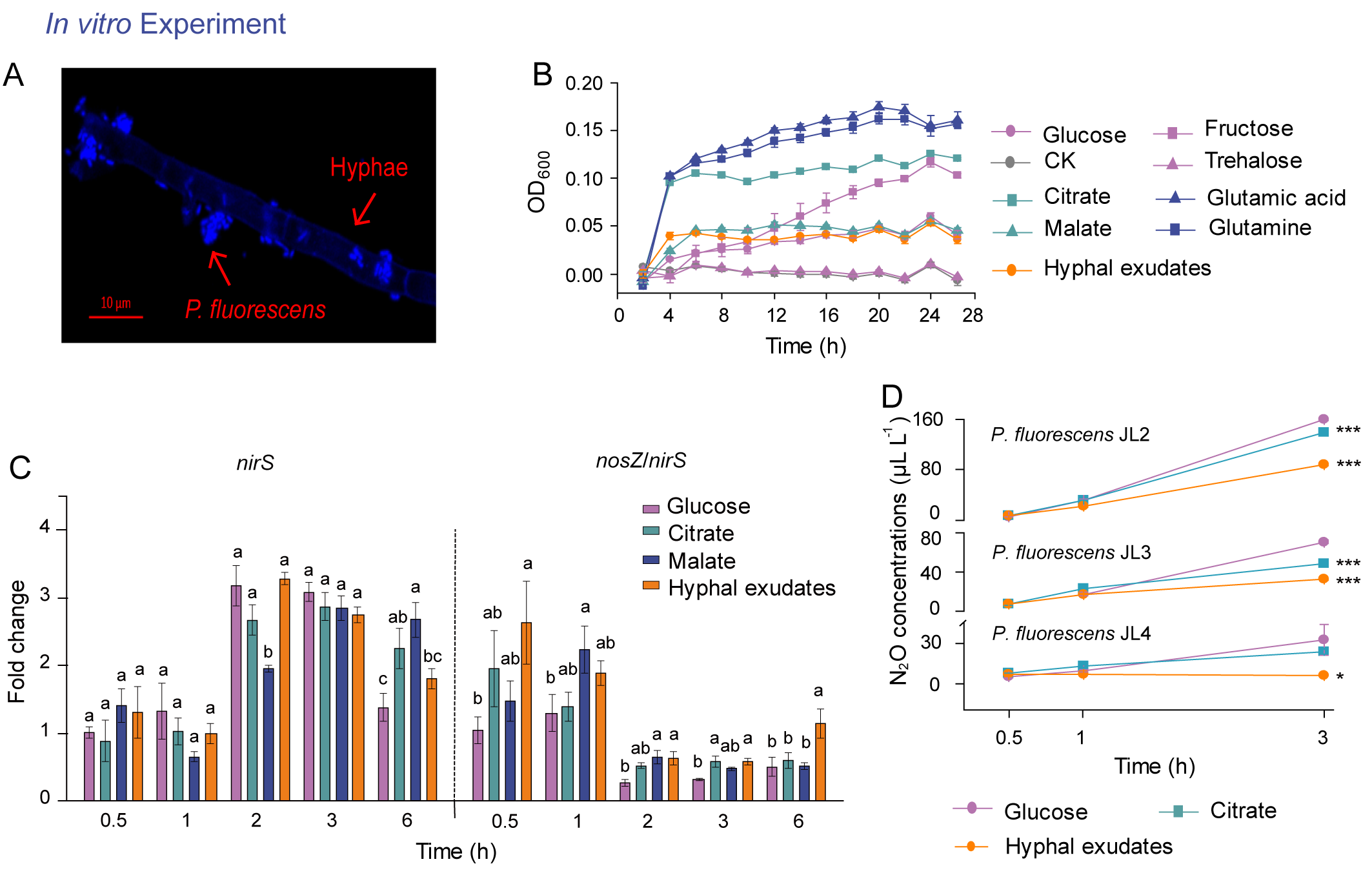

Supplement: Supplementary file 6 — Additional file 5: Fig. S5. Response of Pseudomonas fluorescens to AMF hyphal exudates and major compounds in the in vitro experiment. A, AMF hyphae with attached P. fluorescens stained with 4′,6-diamidino-2-phenylindole (DAPI); scale bar, 10 μm. B, Bacterial optical densities (OD600) of P. fluorescens in response to AMF hyphal exudates and major compounds (n = 3). C, Expression of the nirS gene and nosZ/nirS ratio of P. fluorescens in response to hyphal exudates and major compounds (n = 3). Different lowercase letters indicate significant differences among treatments by the least significant difference (LSD) test at the 5% level. D, Dynamic N2O concentrations in the headspace of serum bottles emitted from three strains of P. fluorescens in response to glucose, citrate, and hyphal exudates (n = 3). Asterisks, significant differences between hyphal exudate or citrate treatment and glucose treatment at 3 h within each strain according to two-tailed unpaired t-test (*, P < 0.05; ***, P < 0.001) . [file 40168_2023_1466_MOESM5_ESM.tif]

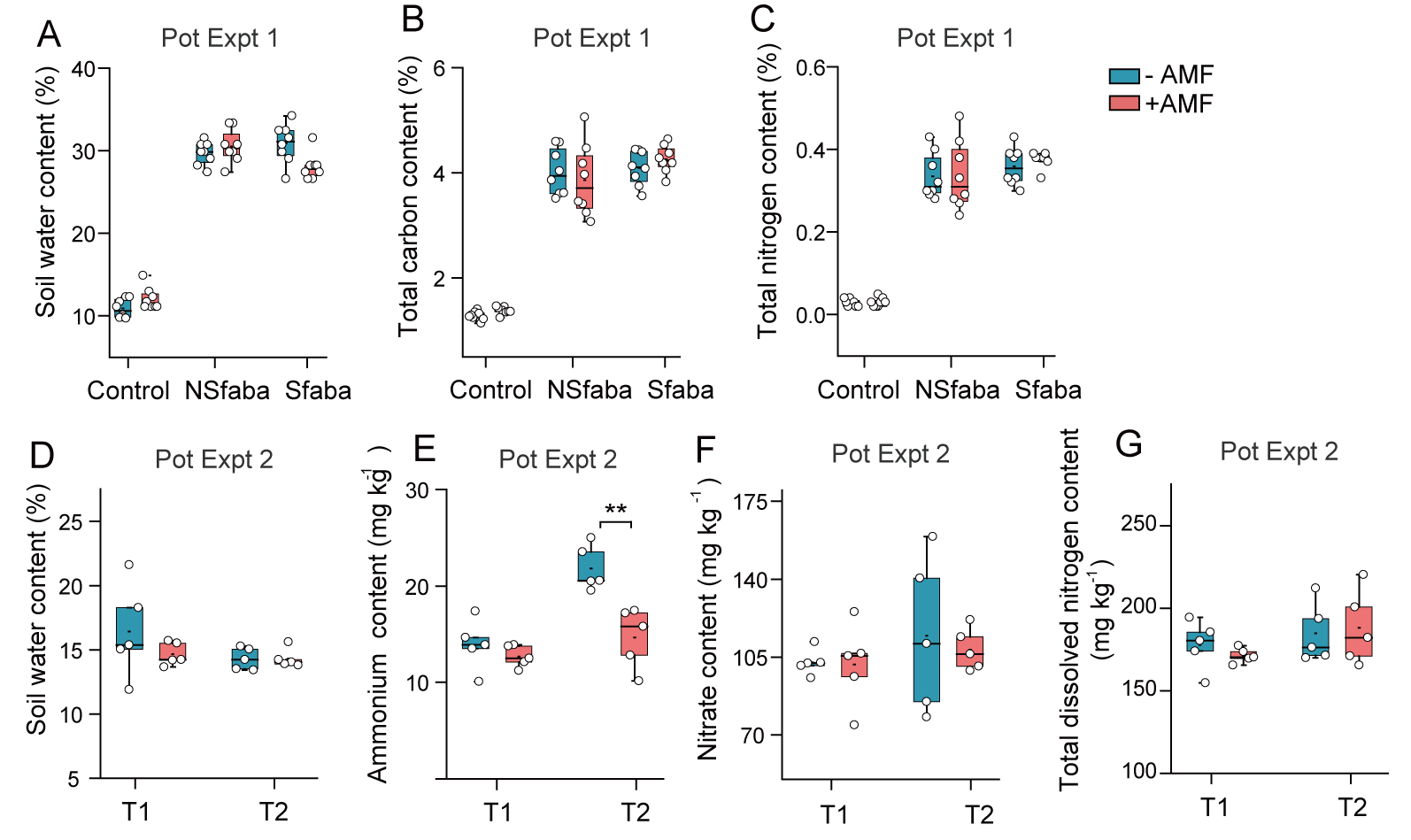

Supplement: Supplementary file 7 — Additional file 6: Fig. S6. Soil water content, total carbon and nitrogen contents, mineral nitrogen and dissolved total nitrogen contents in patches in the absence or presence of AMF A-C, pot expt 1. Soil water content (A), total carbon (B) and nitrogen (C) contents under the −AMF and +AMF treatments in different patches (n = 8). Control, soil patch; NSfaba and Sfaba, patches with unsterilized (NS) or sterilized (S) faba bean residues, respectively. D-G, pot expt 2. Soil water content (D), ammonium (E), nitrate (F) and dissolved total nitrogen (G) contents under the −AMF and +AMF treatments at both harvests (n = 5). T1 and T2, the first (day 24) and second (day 34) harvests, respectively; Asterisks, significant differences between −AMF and +AMF treatments at each harvest (pot Expt 2) according to two-tailed unpaired t-test (*, P < 0.05; **, P < 0.01; ***, P < 0.001). [file 40168_2023_1466_MOESM6_ESM.tif]

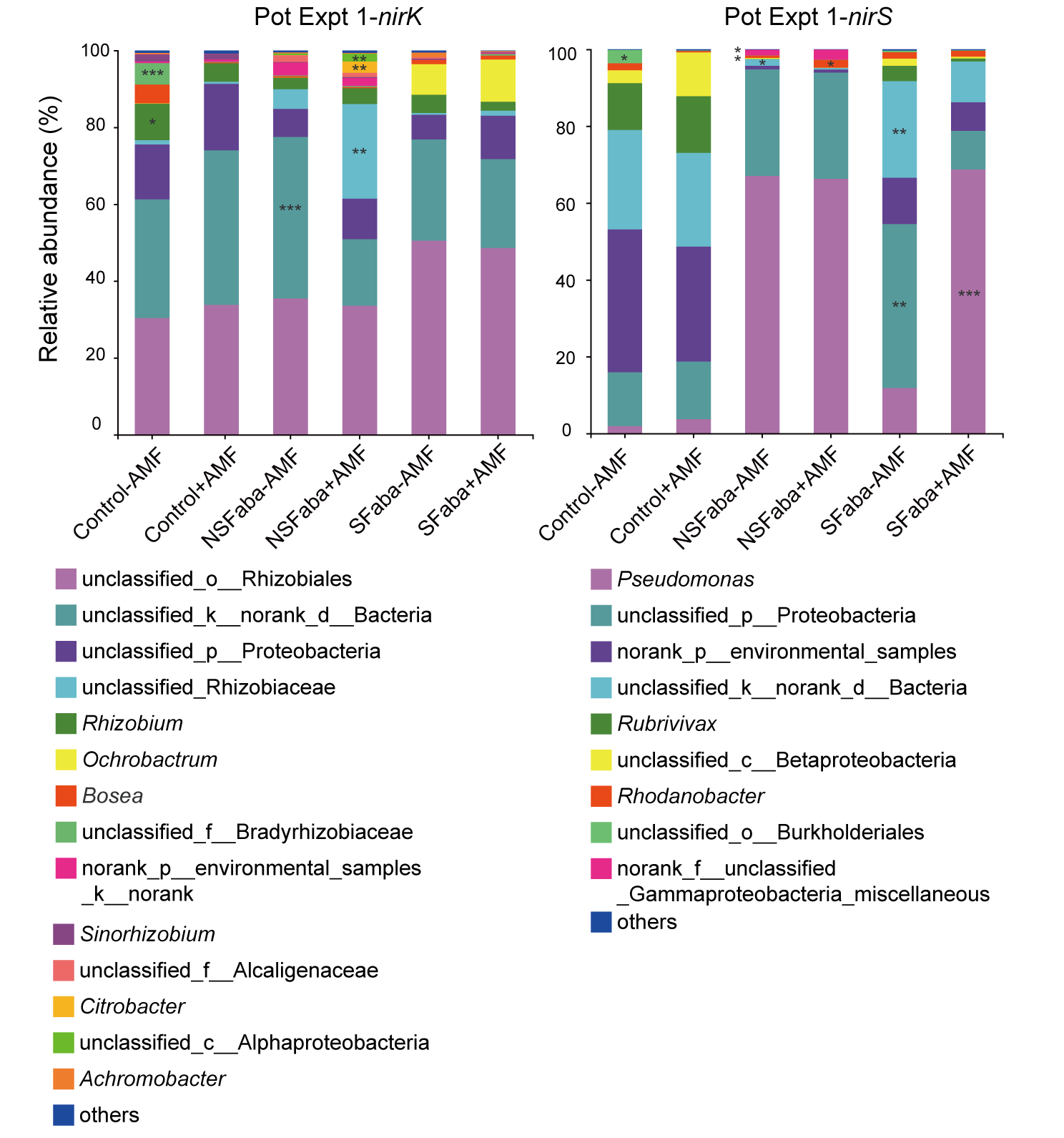

Supplement: Supplementary file 8 — Additional file 7: Fig. S7. Structure of nirK and nirS communities in the absence or presence of AMF. Pot expt 1. Relative abundance of major taxonomic groups of nirK and nirS communities under the −AMF and +AMF treatments in different patches (n = 8). Control, soil patch; NSfaba and Sfaba, patches with unsterilized (NS) or sterilized (S) faba bean residues, respectively; asterisks, significant differences between the −AMF and +AMF treatments in each patch type according to the Wilcoxon rank sum test (*, P < 0.05; **, P < 0.01; ***, P < 0.001). [file 40168_2023_1466_MOESM7_ESM.tif]
