# Supplementary material for: Mycorrhiza-mediated recruitment of complete denitrifying Pseudomonas reduces N2O emissions from soil
Source: Microbiome. 2023 Mar 9;11:45. doi: 10.1186/s40168-023-01466-5 (PMC9996866; doi:10.1186/s40168-023-01466-5)
Supplement: Supplementary file 9 — Additional file 8: Materials and Methods. Supplementary Text. Table S1. Temporal N2O concentrations (μL L-1) in the headspace in the preliminary experiment. Table S2. Primers and PCR conditions used for the PCR. Table S3. Stepwise multiple regression to identify the abundance and expression of key genes involved in N cycling which had the strongest statistical contributions to variation in the cumulative N2O emission in pot expt 2. Independent variables include the abundances and expressions of nirK, nirS and clade I and II nosZ genes. Dependents variable is the cumulative N2O emission. Table S4. Permutational multivariate analysis of variance (PERMANOVA) of the effects of patch type (PT; pot expt 1) or harvest time (HT; pot expt 2) and AMF treatment on microbial communities harbouring nirK, nirS and clade I nosZ based on the gene and transcript sequencing. Table S5. Permutational multivariate analysis of variance (PERMANOVA) of the effect of AMF treatment on clade I nosZ community in different patches (pot expt 1) or harvest time (pot expt 2) based on the gene and transcript sequencing. Table S6. In vitro experiment: metabolite concentrations in the hyphal exudates of Rhizophagus irregularis. Table S7. Effects of patch type and AMF treatment on biomass, N concentration and N content of maize in pot expt 1. Table S8. Effects of harvest time and AMF treatment on biomass, N concentration and N content of maize in pot expt 2. [file 40168_2023_1466_MOESM8_ESM.docx]

Supplementary Information for

Mycorrhiza-mediated recruitment of complete denitrifying *Pseudomonas* reduces N_2_O emissions from soil

**Authors:** Xia Li^1,2^†, Ruotong Zhao^1^†, Dandan Li^1^, Guangzhou Wang^1^, Shuikuan Bei^1^, Xiaotang Ju^3^, Ran An^1^, Long Li^1^, Thomas W. Kuyper^4^, Peter Christie^1^, Franz S. Bender^5^, Ciska Veen^6^, Marcel G.A. van der Heijden^5^, Wim H. van der Putten^6^, Fusuo Zhang^1^, Klaus Butterbach-Bahl^7,8^, Junling Zhang^1^*

Correspondence to: **junlingz@cau.edu.cn**

**This PDF file contains:**

Materials and Methods

Supplementary Text

Supplementary Tables S1 to S8

Materials and Methods

**Supplement to pot [expt](javascript:;) 1**

*Plant growth substrate and AMF inoculum*

*Substrate*: The soil was collected from bare arable land at Quzhou Experimental Station (36°52′ N, 114°01′ E, 40 m a.s.L.) in Quzhou county, Hebei province, north China. The soil is classified as a Cambisol with a silt loam texture. The soil properties were: pH 7.24 (in H_2_O), 7.95 g kg^-1^ organic carbon, 0.90 g kg^-1^ total N, 12.0 mg kg^-1^ Olsen-P, and 176 mg kg^-1^ available K. The soil was air-dried, sieved (˂ 2 mm) and mixed with sand 1:1 (w/w) to serve as growth substrate for the pot experiments. The substrate was γ-irradiated with a maximum dose of 32 kGy to eliminate AMF.

*AMF inoculum*: The root chamber was inoculated with the mycorrhizal fungus *Funneliformis mosseae* (HK01). The inoculum was obtained from the Chinese Bank of Glomeromycota, the Academy of Agricultural and Forestry Science, Beijing. It consisted of spores, mycelium and fine root segments propagated in a 1:1 (w/w) mixture of soil and sand with *Zea mays* L. grown for 4 months in a greenhouse. The inoculum contained approximately 50 spores per gram.

*Preparation of Vicia faba L. residues*

Four *Vicia faba* seeds were planted in a 2-kg pot with the unsterilized growth substrate. Plant growth was ensured by adding sufficient nutrients, namely 100 mg kg^-1^ N (Ca (NO_3_)_2_·4H_2_O), 20 mg kg^-1^P (KH_2_PO_4_) and 100 mg kg^-1^ K (K_2_SO_4_). Soil moisture was controlled at 60% of WFPS with deionized water. After plants were grown for two months in the greenhouse, the belowground parts were harvested and rinsed with deionized water for use.

*Gas analysis*

The stainless-steel tube was sealed on the top using a rubber tube to form a gas port for subsequent gas sampling. Five mL gas were taken from the gas probe using a syringe 24, 48, 72 and 120 h after N addition. The concentrations of N_2_O were determined immediately by gas chromatography (model 7890A, Agilent, Santa Clara, CA) with an electron capture detector.

**Supplement to pot [expt](javascript:;) 2**

*Microcosm set-up*

We enlarged the patch effect by modifying the pot size to avoid gas diffusion (Fig. 1). The volume of the root chamber was 4.5 × 10 × 10 cm^3^, containing 450 g sterilized substrate and 50 g of inoculum. The hyphal chamber was modified to 10 × 10 × 10 cm^3^ only, containing 700 g sterilized substrate. A 330-mL plastic bottle was fixed in the center of the hyphal chamber to reserve space for subsequent addition of the patches. A rectangular window (4 × 6 cm^2^) was cut in the side of the bottle, opened towards the root chamber and covered with a 30-μm mesh to form patches. The bottle had an airtight lid for subsequent gas sampling.

**Plant harvest and determination of soil physicochemical properties**

In both pot experiments, shoots and roots of the maize plants were collected. Patch soil was collected and divided into three portions, one of which was stored at 4 °C for the determination of soil water content, dissolved organic carbon (DOC), total dissolved nitrogen (TDN) and mineral N concentrations. The second portion was stored at -80 °C for molecular analysis and the remainder was stored at room temperature for the determination of AMF hyphal length density (HLD), total carbon (TC) and total nitrogen (TN) contents. Soil gravimetric water content was determined by oven-drying. DOC and TDN in the extracts were determined by infrared spectrometry after combustion at 850 °C [1] . Fresh soil samples were extracted immediately with 0.01 mol L^-1^ CaCl_2_ solution and ammonium (NH_4_^+^-N) and nitrate (NO_3_^−^-N) were determined by continuous flow analysis (Traacs 2000, Bran and Luebbe, Norderstedt, Germany). HLD was quantified by a modified aqueous extraction and membrane-filter technique [2]. Dried shoots and roots of maize and soil samples were ground in a ball mill, TC and TN concentrations were determined with an elemental analyzer (vario Macro cube, Elementar Analysensysteme GmbH, Langenselbold, Germany).

**DNA and RNA extraction, cDNA synthesis,** **real-time PCR, high-through sequencing and shotgun metagenomic sequencing**

Soil DNA and RNA were extracted according to the manufacturers’ instructions. The DNA and RNA quality was determined with a NanoDrop ND-2000 (Thermo Fisher, Waltham, MA). The real-time quantitative PCR (qPCR) of *nirK*, *nirS*, and *nosZ* (clade I and Ⅱ) genes were conducted using QuantStudio 6 Flex (Applied Biosystems, Waltham, MA). The 10 μL qPCR reactions consisted of 5 μL of SYBR Premix ExTaq II (2×) (TaKaRa Bio, Kusatsu, Shiga, Japan), 0.2 μL of ROX Reference Dye II, 0.25 of μL of each primer (10 μM), 3.3 μL of ddH_2_O, and 1 μL of template DNA or cDNA. Each reaction was prepared in triplicate. The standard curves of each gene were obtained using a 10-fold serial dilution series (10^8^-10^2^ copies) of known copy numbers of the plasmid DNA-containing target gene [3]. The amplification efficiencies were 90-105% and the *R*^2^ value of the standard curves was 0.99-1.00 for all genes.

The microbial communities harboring the key genes *nirK*, *nirS* and clade I *nosZ* were determined. Paired-end sequencing (2 × 300) was conducted through Illumina MiSeq PE amplicon sequencing by Majorbio Bio-Pharm Technology Co. Ltd., Shanghai, China. The key genes were amplified using the primer pairs as in qPCR. Each unique barcode sequence was added to the forward and reverse primers to each sample. The PCR reactions were conducted using a thermal cycler (Bio-Rad, Hercules, CA) and the detailed thermal conditions are shown in Table S2. All PCR reactions were conducted in duplicate and contained 44 μL of Golden Star T6 Super PCR Mix (1.1×) (Tsingke, Beijing, China), 2 μL of each primer (10 μM), and 2 μL of DNA or cDNA template in a total volume of 50 μL. Duplicate PCR products were pooled and purified. These purified PCR products of each key gene were combined in equimolar ratios to obtain a quantitative sample DNA library that was then sequenced according to the manufacturer’s instructions. The raw sequencing reads were demultiplexed and quality-filtered by fastp version 0.20.0 [4] then merged by Flash version 1.2.7 [5]. The Uparse program was used to remove chimeras and classify the sequences into operational taxonomic units (OTUs) at 97% similarity level [6, 7]. The taxonomy of each OTU representative sequence was analyzed with RDP Classifier version 2.2 against the FGR database from GenBank using a confidence threshold of 0.7 [8]. For fair comparison, subsamples were created according to the minimum sequence number.

To further explore the changes of potential microbial functions in response to AMF, we analyzed the metagenomes (MGs) of the soil samples of T2 in pot expt 2. DNA samples were fragmented to an average size of approximately 400 bp using Covaris M220 (Gene Company Limited, China). Paired-end library was constructed using NEXTFLEX Rapid DNA-Seq (Bioo Scientific, Austin, TX, USA), sequencing was performed on Illumina NovaSeq with a paired-end protocol (Illumina Inc., San Diego, CA, USA). The low-quality reads were filtered by fastp [4], the high-quality reads were assembled with MEGAHIT [9]. The open reading frames (ORF) were predicted using MetaGene [10] and clustered with a 0.95 similarity threshold to generate non-redundant gene catalog using CD-HIT version v4.8.1. The high-quality reads were compared with non-redundant gene sets with a 0.95 identity using SOAAPaligner [11]. Representative sequences of non-redundant gene catalog were compared against to KEGG (the Kyoto Encyclopedia of Genes and Genomes) database and NCBI NR database with e-value cutoff of 1e^-5^ using BLASTP Version 2.2.28+ [12]. In this study, the nitrous-oxide reductase (K00376) and the pathways associated with carbon metabolism and citrate cycle were selected.

**Supplement to the collection and analysis of** **hyphal exudates**

Petri dishes (90 × 15 mm) were split into two chambers with a plastic barrier in the center. One chamber (root chamber, RC) was for both root and hyphal growth and the other chamber (hyphal chamber, HC) was for hyphal growth. Twenty-five mL MSR medium [13] with 3 g L^-1^ phytagel were poured in the RC and 5 mm medium was removed with a sterilized scalpel along the central barrier to prevent diffusion of carbon from RC to HC. Then 4 mL MSR medium with 3 g L^-1^ phytagel minus sucrose, NaFeEDTA and vitamin sources to eliminate carbon sources except exudates was added in a slope from top to bottom of the plastic barrier in the HC to form a slope in order to ensure hyphal proliferation to the hyphal chamber. Excised transformed carrot roots associated with *Rhizophagus irregularis* was transferred in the RC and incubated at 27°C in the dark. After two months, when the slope in HC was covered by AMF hyphae, 10 mL of liquid MSR medium minus sucrose, NaFeEDTA and vitamin sources was added to the HC. After one further month, when most of the liquid MSR medium was covered by actively growing AMF hyphae, the solution containing hyphal exudates was collected and passed through an Acrodisc syringe filter (0.22-μm Supor Membrane, Pall Corporation, Port Washington, NY) to remove AMF hyphae. The exudates were immediately frozen at -80 °C for subsequent analysis. The analysis of carboxylates and amino acids in hyphal exudates was conducted using an Agilent 7890A gas chromatography system coupled to an Agilent 5975C inert MSD system (Agilent Technologies, Santa Clara, CA). The analysis of sugars in hyphal exudates was conducted using a UHPLC system (Vanquish, Thermo Fisher Scientific, Waltham, MA) with a UPLC BEH Amide column coupled to Q Exactive HFX mass spectrometer (Orbitrap MS, Thermo Fisher).

**Supplement to the serum bottle assay**

The *P. fluorescens* JL1 suspension was prepared as follows. Bacteria were cultured in liquid LB medium with shaking at 180 rpm for 12 h at 30 °C. To eliminate the effect of carbon source in LB medium, the medium was then centrifuged at 5000 × g for 6 min. The supernatant was discarded and the pellet was re-suspended and washed with phosphate buffered solution (PBS) three times and adjusted to an OD_600_ value of 4. Then 1 mL suspension was centrifugated and the supernatant was discarded. The pellet was re-suspended with 10 mL modified CB medium and transferred into a 120-mL anaerobic serum bottle. The headspace of the serum bottles was flushed with pure N_2_ for 5 min to create anaerobic conditions. After equilibrating the air pressure, all serum bottles were shaken at 180 rpm and maintained at 30 °C. Eight-mL gas samples in the headspace of the serum bottles were collected using a sterilized gas-tight syringe for the detection of N_2_O. Then 8 mL of N_2_ were replenished quickly to balance the air pressure in the bottles.

**Supplement to the assay of *nirS* and *nosZ* gene expression**

Total RNA was extracted from 1 ml culture with an RNAprep pure cell/bacteria kit and treated with DNase I (Tiangen, Beijing) and then quantified with NanoDrop (Thermo Fisher Scientific). One μg of total RNA was reverse-transcribed with a PrimeScript RT reagent kit with gDNA eraser (Perfect Real Time) (TaKaRa Bio) according to the manufacturer’s instructions. Relative changes in *nirS* and *nosZ* genes were normalized by the 2^−ΔΔCt^ method [14], referring to the ΔCt value in the glucose treatment harvested at the 0.5-h sampling time. The constitutively expressed RNA polymerase sigma factor *RpoD* was used as a house-keeping gene.

**Genomic sequencing of *P. fluorescens***

The draft genomes of *Pseudomonas fluorescens* strains (JL1, JL2 and JL3) were sequenced using an Illumina NovaSeq 6000 platform (Majorbio BioTech Co., Ltd., Shanghai, China) and generated 2 × 150 bp paired-end libraries (average read length of ~ 400 bp). The sequenced reads were assembled using SOAPdenovo [15]. Glimmer was used for coding sequence (CDS) prediction. The predicted CDSs were annotated from the KEGG database using BLASTP and Diamond. Each set of query proteins was aligned with the databases, and annotations of best-matched subjects (e-value < 10^-5^) were obtained for gene annotation.

**N_2_O production by three other *P. fluorescens* strains**

Three other *nosZ* type *P. fluorescens* strains (JL2, JL3 and JL4) isolated from patches at the second harvest of pot expt 2 were randomly selected to validate the N_2_O production. We selected citrate and hyphal exudates as carbon sources, and glucose was used as the control. The experiment procedure was the same as in the *serum bottle assay*. The gas was measured after 0.5, 1 and 3 hours and there were 3 replicates of each treatment.

**Microscopic observation of *P. fluorescens* on hyphal surfaces**

The two-chamber Petri dishes were used to collect hyphal exudates. When the HC was covered by actively growing hyphae, the suspension of *P. fluorescens* JL1 was added with a final OD_600_ value of 0.2-0.4. After culturing for 12 hours the hyphae were washed with PBS and fixed in 4% paraformaldehyde for 30 minutes. The hyphae were stained with 1 mg ml^-1^ 4′,6-diamidino-2-phenylindole (DAPI) for 15 min in the dark. The slides were imaged by confocal microscopy using a Carl Zeiss LSM 880 system (Carl Zeiss AG, Oberkochen, Germany).

**Supplement to the statistical analysis**

All data were first tested for normality and homogeneity of variance before analysis of variance (ANOVA) and tested for normality before *t*-tests and Pearson correlation analysis. The key gene and transcript copies were log_10_(*x*)-transformed to fulfil normality and homogeneity of variance. In pot expt 1 the differences in N_2_O concentration between –AMF and +AMF treatments within each patch type at each gas-sampling time after supplementation with NO_3_^−^-N or NH_4_^+^-N were evaluated by two-tailed unpaired *t*-tests. Except for patch N_2_O concentrations, N form had no significant effect on the variables and therefore was not considered in further analysis. The differences in key gene copies, HLD and soil physicochemical properties between –AMF and +AMF treatments within each patch type were evaluated by two-tailed unpaired *t*-tests. In pot expt 2 the differences in N_2_O flux, cumulative emissions, key gene and transcript copies, HLD and physicochemical properties between –AMF and +AMF treatments within each harvest time (or gas-sampling time) were also evaluated with two-tailed unpaired *t*-tests. In pot expts 1 and 2 the relationships between N_2_O concentrations or cumulative emissions, *nosZ* gene and transcript copies, HLD and DOC contents were examined using Pearson correlation analysis. Stepwise multiple regression was used to determine the variation in cumulative N_2_O emissions that could be explained by the abundance and expression of key genes involved in N cycling, with a criterion of *P < 0.05* to accept variables.

In pot expts 1 and 2 the effects of patch type (harvest time) and AMF treatment on microbial community were analyzed using permutational multivariate analysis of variance (PERMANOVA) conducted using the Vegan package [16]. Linear discriminant analysis (LDA) effect size (LEfSe) was conducted to discover high-dimensional biomarkers and identify significantly different taxa in microbial communities between –AMF and +AMF treatments within each patch type. For LEfSe, Kruskal-Wallis and pairwise Wilcoxon tests were conducted followed by LDA to assess the effect size of each differentially abundant taxon [17]. In pot expt 2, Spearman correlation analysis was used to estimate the relationships between cumulative N_2_O emission and the relative abundance of the N_2_O-reducing *Pseudomonas* at gene and transcript levels. In both pot experiments and the *in vitro* experiment, the difference in the relative abundance of major genera and species of denitrifying bacteria between –AMF and +AMF treatments was tested using Wilcoxon rank sum tests.

In the *in vitro* experiment the effects of carbon source on bacterial OD_600_, N_2_O concentrations, expressions of *nirS* and *nosZ* genes and the *nosZ*/*nirS* ratio at each sampling time and chemotaxis area were determined by least significant difference (*LSD*) test at the 5% level after one-way ANOVA. Nonmetric multidimensional scaling (NMDS) plots were generated using the ggplot2 package to visualize the variation in microbial community among the different samples based on Bray-Curtis dissimilarity [18]. In the inoculation experiment the cumulative N_2_O emissions and CFU were log_10_(*x*)-transformed to fulfil normality and homogeneity of variance. The effects of different treatments on patch N_2_O fluxes at each sampling time, cumulative N_2_O emissions, bacterial CFU, expressions of *nirS* and *nosZ* genes and the ratio of *nosZ*/*nirS* were determined by least significant difference (LSD) test at the 5% level after one-way ANOVA.

In the field experiment, the differences in HLD and *nosZ* gene copies between monoculture and intercropping treatments within each P application rate were evaluated using two-tailed unpaired *t* tests. The relationships between *nosZ* gene copies and HLD were examined using Pearson correlation analysis.

All of the above parametric analyses were conducted using the SPSS version 22.0 software package (SPSS, Chicago, IL). All nonparametric tests were conducted in R (version 4.0.3).

Supplementary Text

**Hyphal length density and physicochemical properties in the residue patches**

In pot expt 1, water content, TC and TN contents in organic patches were 2.59, 3.05 and 11.83 times higher than those in soil patches, respectively. The values were not significantly affected by AMF (Fig. S6A-C).

In pot expt 2, AMF significantly increased patch DOC and TN contents and reduced the NH_4_^+^-N content at the second harvest. In the absence of AMF, the patch NH_4_^+^-N content was higher at the second than at the first harvest, while patch TN content showed the opposite trend (Figs. S1C, F and S6E). Patch water content, NO_3_^−^, TDN and TC contents were not significantly affected by harvest time or AMF treatment (Figs. S1E and S6D, F, G).

**Structure of *nirK* and *nirS* communities**

In pot expt 1, at the genus level, *nirK* community comprised mainly *Rhizobium*, *Ochrobactrum*, *Bosea*, *Sinorhizobium*, *Citrobacter* and *Achromobacter*, accounting for 4-17% of the total sequences. The *nirS* community comprised mainly *Pseudomonas*, *Rubrivivax*, and *Rhodanobacter*, accounting for 16-71% of the total sequences (Fig. S7). Permutation multiple variance analysis (PERMANOVA) showed that the structure of *nirK* and *nirS* communities was significantly affected by patch type but not by AMF (Table S4). For *nirK* community, *Sinorhizobium*, *Rhizobium*, *Afipia* and *Bradyrhizobium* were mainly enriched in patches with soil. *Citrobacter* was mainly enriched in the NSfaba patches. *Ochrobactrum*, *Starkeya* and *Achromobacter* were mainly enriched in the Sfaba patches. For *nirS* community, *Aromatoleum*, *Cupriavidus* and *Rubrivivax* were mainly enriched in the patches with soil. *Pseudomonas*, *Ralstonia* and *Bordetella* were mainly enriched in the NSfaba patches, in which *Pseudomonas* was the dominant genus, accounting for 66% of *nirS* community (Fig. S7).

For *nirK* community, AMF significantly reduced the relative abundance of *Rhizobium* in soil patches and increased the relative abundance of *Citrobacter* and an unclassified genus affiliated with the Rhizobiaceae in the NSfaba patches. For *nirS* community, AMF significantly increased the relative abundance of *Rhodanobacter* and decreased the relative abundance of *Rubrivivax* in the NSfaba patches, but did not alter the relative abundance of *Pseudomonas*. By contrast, AMF significantly increased the relative abundance of *Pseudomonas* in the Sfaba patches (Fig. S7).

In pot expt 2, at the genus level, *nirK* community comprised mainly *Rhizobium* and *Achromobacter* (only at transcript level), accounting for 5-6% (at gene level) and 4-6% (at transcript level) of the total sequences (relative abundance ≥ 1%). The *nirS* community comprised mainly *Pseudomonas*, accounting for 30-43% (at gene level) and 47-62% (at transcript level) of the total sequences. At the first harvest, AMF altered the community structure of *nirK* community by significantly increasing the relative abundance of genera from the Rhizobiales at the gene level, and *Sinorhizobium* and *Achromobacter* at the transcript level. At the second harvest, AMF decreased the relative abundance of *Bosea* at the gene level, but did not alter the community structure of *nirK* community at either gene or transcript level (Fig. S3A, B). At the first harvest, AMF altered the structure of *nirS* community only at the gene level by significantly reducing the relative abundance of *Rhodanobacter*. At the second harvest, AMF changed the structure of *nirS* community by significantly increasing the relative abundance of genera from the γ-proteobacteria at transcript level (Fig. S3A, B).

***In vitro* experiment: cultivation of denitrifying microbes**

In total, 129 isolates were obtained, and they were classified into 27 different species based on nearly full 16S rRNA genes (~1500 bps). The abundant species was defined as those comprised more than 1% of the total isolates (i.e., ≥ 2 isolates). Among them, 12 species were detected as single isolates and considered as rare species and not further analyzed. The 15 abundant species were affiliated with *Pseudomonas* (5 species), *Paenarthrobacter*, *Agrobacterium*, *Rhodococcus*, *Arthrobacter* (2 species), *Achromobacter*, *Variovorax*, *Rhizobium*, *Microbacterium* and *Massilia* (Fig. S4A, B). *Pseudomonas* was the most abundant genus, accounting for 34% of the population of abundant-species isolates (Fig. S4B). AMF significantly altered the community structure of culturable denitrifiers (Fig. S4C) by increasing the relative abundance of *Pseudomonas* while decreasing that of *Arthrobacter* (Fig. S4B).

**Metabolites detected in the hyphal exudates of *R. irregularis***

In the *in vitro* experiment the total carbon concentration in the hyphal exudates was ~ 7.16 mM. The dominant carbohydrates in the hyphal exudates were glucose (17.49 μM), fructose (10.21 μM) and trehalose (9.67 μM). The dominant carboxylates were malate (8.00 μM) and citrate (7.13 μM), and dominant amino acids were glutamine (42.92 μM) and glutamic acid (8.55 μM) (Table S6).

**Expression of *nirS* gene of the representative strain and N_2_O emissions of three other strains of *P. fluorescens***

In the *in vitro* experiment, the expression of the *nirS* gene of *P. fluorescens* JL1 did not differ among treatments except in the malate treatment at 2 h and also in the treatment of carboxylates at 6 h (Fig. S5C). In order to examine whether the effect of hyphal exudates and citrate on the N_2_O emission of *Pseudomonas* was universal, we randomly selected three other strains of *P*. *fluorescens* (JL2, JL3 and JL4) carrying the *nosZ* gene. The treatment effect was significant at 3 h. The addition of hyphal exudates significantly reduced N_2_O emissions in the treatment of all three strains, and the addition of citrate significantly reduced N_2_O emissions in the strains of JL2 and JL3 (but not JL4) compared to glucose addition (Fig. S5D).

**Biomass, nitrogen concentrations and contents of maize**

In pot expt 1, AMF significantly increased the dry weight and shoot and root nitrogen contents of maize in all patches. The root N concentrations of maize was increased only in the NSfaba patches. Moreover, AMF significantly decreased shoot N concentrations of maize (Table S7).

In pot expt 2, AMF significantly increased the root dry weight of maize only at the second harvest. At both harvests shoot dry weight, and N concentrations and contents of maize shoots and roots were not significantly affected by AMF (Table S8).

|  | 0 h | 2 h | 3 h | 4 h | 6 h |
| --- | --- | --- | --- | --- | --- |
| Repeat 1 | 0.397 | 0.685 | 0.728 | 1.187 | 1.281 |
| Repeat 2 | 0.354 | 0.528 | 0.693 | 0.796 | 0.921 |
| Repeat 3 | 0.351 | 0.595 | 0.764 | 0.917 | 0.983 |

Table S1 Temporal N_2_O concentrations (μL L^-1^) in the headspace in the preliminary experiment

Table S2 Primers and PCR conditions used for the PCR

| Target gene | Primer | Sequence (5′-3′) | Thermal profile (PCR) | Thermal profile (qPCR) | Reference |
| --- | --- | --- | --- | --- | --- |
| *nirK* | F1aCu | ATCATGGTSCTGCCGCG | 3 min at 94°C, 35 cycles consisting of 30 s at 94°C, 30 s at 61°C and 1 min at 72°C, 10 min at 72°C. | 30 s at 95°C, 44 cycles consisting of 5 s at 95°C, 35 s at 60°C and 30 s at 72°C, 10 s at 95 °C. | [19] |
|  | R3Cu | TTGGTGTTRGACTAGCTCCG |  |  |  |
| *nirS* | Cd3aF | GTSAACGTSAAGGARACSGG | 10 min at 95°C, 40 cycles consisting of 30 s at 94.5°C, 20 s at 57°C and 20 s at 72°C, 5 s at 72°C. | 30 s at 95°C, 44 cycles consisting of 5 s at 95°C, 45 s at 55°C and 45 s at 72°C, 10 s at 95 °C. | [3, 19] |
|  | R3cd | GASTTCGGRTGSGTCTTGA |  |  |  |
| clade I *nosZ* | *nosZ*2F | CGCRACGGCAASAAGGTSMSSGT | 5 min at 95°C, 40 cycles consisting of 60 s at 95°C, 1 min at 60°C and 1 min at 72°C, 10 min at 72°C. | 30 s at 95°C, 44 cycles consisting of 5 s at 95°C, 35 s at 60°C and 30 s at 72°C, 10 s at 95 °C. | [20] |
|  | *nosZ*2R | CAKRTGCAKSGCRTGGCAGAA |  |  |  |
| clade II *nosZ* | *nosZ*-II-F | CTIGGICCIYTKCAYAC | **\** | 2 min at 95°C, 40 cycles consisting of 15 s at 95°C, 15 s at 55°C and 20 s at 72°C, 5 s at 95 °C. | [21] |
|  | *nosZ*-II-R | GCIGARCARAAITCBGTR C |  |  |  |
| *nosZ* | *nosZ* 1527F | CGCTGTTCHTCGACAGYCA | 30 s at 95°C, 44 cycles consisting of 5 s at 95°C, 35 s at 60°C and 30 s at 72°C, 10 s at 95 °C. | 30 s at 95°C, 44 cycles consisting of 5 s at 95°C, 35 s at 60°C and 30 s at 72°C, 10 s at 95 °C. | [22] |
|  | *nosZ*1773R | ATRTCGATCARCTGBTCGTT |  |  |  |
| 16S | 27F | AGAGTTTGATCCTGGCTCAG | 3 min at 94°C, 24 cycles consisting of 30 s at 94°C, 30 s at 54°C and 90 s at 72°C. | **\** | [23] |
|  | 1492R | TACGGYTACCTTGTTACGACTT |  |  |  |

Table S3 Stepwise multiple regression to identify the abundance and expression of key genes involved in N cycling which had the strongest statistical contributions to variation in the cumulative N_2_O emission in pot expt 2. Independent variables include the abundances and expressions of *nirK*, *nirS* and clade I and II *nosZ* genes. Dependents variable is the cumulative N_2_O emission.

| Dependents | Period | Variables related | *R*^2^ (model) | *F* (model) | *P* (model) | Variables removed |
| --- | --- | --- | --- | --- | --- | --- |
| Cumulative N_2_O emissions | T1 | *nirK* gene expression | 0.72 | 23.72 | 0.001 | Abundances of *nirK*, *nirS* and clade I and II *nosZ* genes; expression of *nirS*, clade I and II *nosZ* genes |
|  | T2 | *nosZ* I gene expression | 0.61 | 15.04 | 0.005 | Abundances of *nirK*, *nirS* and clade I and II *nosZ* genes; expression of *nirK*, *nirS* and clade II *nosZ* genes |

| Variable | expt 1 | | | | | | expt 2 | | | | | | | |
| --- | --- | --- | --- | --- | --- | --- | --- | --- | --- | --- | --- | --- | --- | --- |
|  | PT | | AMF | | PT * AMF | | level | HT | | AMF | | HT * AMF | | |
|  | *R*^2^ | *P* | *R*^2^ | *P* | *R*^2^ | *P* |  | *R*^2^ | *P* | *R*^2^ | *P* | *R*^2^ | *P* |  |
| *nirK* | 0.199 | **0.001** | 0.024 | 0.162 | 0.032 | 0.079 | DNA | 0.108 | **0.038** | 0.136 | **0.014** | 0.085 | 0.069 |  |
|  |  |  |  |  |  |  | cDNA | 0.198 | **0.001** | 0.113 | **0.004** | 0.047 | 0.288 |  |
| *nirS* | 0.138 | **0.003** | 0.022 | 0.262 | 0.034 | 0.103 | DNA | 0.077 | 0.110 | 0.136 | **0.008** | 0.050 | 0.353 |  |
|  |  |  |  |  |  |  | cDNA | 0.075 | 0.090 | 0.107 | **0.006** | 0.042 | 0.591 |  |
| clade I *nosZ* | 0.197 | **0.001** | 0.040 | **0.037** | 0.036 | **0.045** | DNA | 0.105 | **0.007** | 0.115 | **0.005** | 0.076 | 0.082 |  |
|  |  |  |  |  |  |  | cDNA | 0.151 | **0.003** | 0.117 | **0.003** | 0.056 | 0.205 |  |

Table S4 Permutational multivariate analysis of variance (PERMANOVA) of the effects of patch type (PT; pot expt 1) or harvest time (HT; pot expt 2) and AMF treatment on microbial communities harbouring *nirK*, *nirS* and clade I *nosZ* based on the gene and transcript sequencing

Values in bold, statistically significant sources of variation.

Table S5 Permutational multivariate analysis of variance (PERMANOVA) of the effect of AMF treatment on clade I *nosZ* community in different patches (pot expt 1) or harvest time (pot expt 2) based on the gene and transcript sequencing

| Variable | expt 1 | | | | | | expt 2 | | | | |  |
| --- | --- | --- | --- | --- | --- | --- | --- | --- | --- | --- | --- | --- |
|  | Control | | NSfaba | | Sfaba | | level | T1 | | T2 | | |
|  | *R*^2^ | *P* | *R*^2^ | *P* | *R*^2^ | *P* |  | *R*^2^ | *P* | *R*^2^ | *P* | |
| clade I *nosZ* | 0.174 | **0.009**** | 0.333 | **0.001***** | 0.185 | **0.002**** | DNA | 0.264 | **0.020*** | 0.169 | **0.090** | |
|  |  |  |  |  |  |  | cDNA | 0.183 | **0.015*** | 0.233 | **0.020*** | |

Control, soil patch; NSfaba and Sfaba, patches with unsterilized (NS) or sterilized (S) faba bean residues, respectively;

Values in bold, statistically significant difference.

Table S6 *In vitro* experiment: metabolite concentrations in the hyphal exudates of *Rhizophagus irregularis*

| Group of compounds | Composition | Carbon concentrations (μmol C L^-1^) |
| --- | --- | --- |
| Sugars | Glucose | 17.49 |
|  | Fructose | 10.21 |
|  | Trehalose | 9.67 |
|  | Cellobiose | 1.67 |
|  | Maltose | 0.54 |
|  | Lactose | 0.43 |
| Carboxylates | Malate | 8.00 |
|  | Citrate | 7.13 |
|  | Lactate | 6.54 |
|  | Malonate | 5.30 |
|  | cis-Aconitate | 3.94 |
|  | Fumarate | 1.81 |
|  | Succinate | 1.51 |
|  | 2-Ketoglutarate | 1.19 |
|  | Glutarate | 0.61 |
|  | Isocitrate | 0.30 |
| Amino acids | Glutamine | 42.92 |
|  | Glutamic acid | 8.55 |
|  | Isoleucine | 7.49 |
|  | Leucine | 5.56 |
|  | Alanine | 4.33 |
|  | Proline | 4.17 |
|  | Serine | 3.85 |
|  | Tyrosine | 2.79 |
|  | Aspartic acid | 2.54 |
|  | Glycine | 1.53 |
|  | Lysine | 1.41 |

Table S7 Effects of patch type and AMF treatment on biomass, N concentration and N content of maize in pot expt 1

| PT | AMF | Biomass (g) | | | | |  | N concentration (mg g^-1^) | | | | | |  | N content (mg) | | | | |
| --- | --- | --- | --- | --- | --- | --- | --- | --- | --- | --- | --- | --- | --- | --- | --- | --- | --- | --- | --- |
|  |  | Shoot | |  | Root | |  | Shoot | |  | | Root | |  | Shoot | |  | Root | |
| Control | –AMF | 5.81*** | B |  | 2.23*** | A |  | 5.38** | B |  | 5.53 | | B |  | 30.94*** | B |  | 12.28*** | B |
|  | +AMF | 10.32 |  |  | 2.87 |  |  | 4.23 |  |  | | 5.55 |  |  | 43.54 |  |  | 15.88 |  |
| NSfaba | –AMF | 6.79* | A |  | 2.46* | A |  | 5.81*** | A |  | | 5.26* | B |  | 39.38** | A |  | 12.90*** | B |
|  | +AMF | 11.18 |  |  | 2.88 |  |  | 4.66 |  |  | | 5.66 |  |  | 51.99 |  |  | 16.29 |  |
| Sfaba | –AMF | 6.94*** | AB |  | 2.26*** | A |  | 5.26** | B |  | | 5.9 | A |  | 36.34* | B |  | 13.32*** | A |
|  | +AMF | 10.03 |  |  | 3.19 |  |  | 4.41 |  |  | | 5.81 |  |  | 43.99 |  |  | 18.42 |  |
| ANOVA^2^ | | | | | | | | | | | | | | | | | | | |
| Source of variation | | *F* | *P* |  | *F* | *P* |  | *F* | *P* |  | | *F* | *P* |  | *F* | *P* |  | *F* | *P* |
| PT | | 2.82 | **0.071** |  | 1.62 | 0.211 |  | 4 | **0.026** |  | | 4.6 | **0.016** |  | 8.64 | **<0.001** |  | 7.47 | **0.002** |
| AMF | | 159.12 | **<0.001** |  | 64.54 | **<0.001** |  | 56.46 | **<0.001** |  | | 1.01 | 0.321 |  | 42.32 | **<0.001** |  | 106.96 | **<0.001** |
| PT × AMF | | 2.06 | 0.14 |  | 3.3 | **0.047** |  | 0.5 | 0.61 |  | | 1.78 | 0.182 |  | 0.96 | 0.391 |  | 1.91 | 0.16 |

^1^Values are means ± standard error, n = 8; Control, soil patch; NSfaba and Sfaba, patches with unsterilized (NS) or sterilized (S) faba bean residues, respectively; –AMF and +AMF, without or with mycorrhizal inoculation, respectively; Asterisk, significant difference between –AMF and +AMF treatments within each patch type according to *t*-tests (*, *P* < 0.05; ***P* < 0.01; ***, *P* < 0.001); Different capital letters, significant differences among different patch types at *P* < 0.05.

^2^ Values in bold, statistically significant sources of variation according to analysis of variance (ANOVA).

| HT^1^ | AMF | Biomass (g) | | | | | |  | N concentration (mg g^-1^) | | | | | |  | | N content (mg) | | | | | | | |  | | |
| --- | --- | --- | --- | --- | --- | --- | --- | --- | --- | --- | --- | --- | --- | --- | --- | --- | --- | --- | --- | --- | --- | --- | --- | --- | --- | --- | --- |
|  |  | Shoot | | |  | Root | |  | Shoot | | |  | Root | |  | | Shoot | | |  | | Root | | |  | |  |
| T1 | –AMF | 5.4 | B | |  | 1.88 | B |  | 11.6 | | A |  | 10.0 | A |  | | 62.61 | | B |  | | 18.72 | A |  | |  |  |
|  | +AMF | 5.19 |  |  |  | 1.91 |  |  | 11.8 | |  |  | 10.2 |  |  | | 60.71 | |  |  | | 19.51 |  |  | |  |  |
| T2 | –AMF | 7.12 | A | |  | 2.41* | A |  | 12.4 | | A |  | 8.2 | B |  | | 87.46 | | A |  | | 19.69 | A |  | |  |  |
|  | +AMF | 7.42 |  |  |  | 3.51 |  |  | 12.6 | |  |  | 6.6 |  |  | | 93.11 | |  |  | | 22.51 |  |  | |  |  |
|  | | | | ANOVA^2^ | | | | | |  | | | | | |  |  |  |  |  |  |  |  |  |  |  |  |
| Source of variation | | *F* | *P* | |  | *F* | *P* |  | *F* | | *P* |  | *F* | *P* | | |  | *F* | *P* | |  | *F* | *P* | | |  |  |
| HT | | 30.78 | **<0.001** | |  | 32.08 | **<0.001** |  | 1.05 | | 0.32 |  | 20.67 | **<0.001** | | |  | 32.48 | **<0.001** | |  | 1.81 | 0.197 | | |  |  |
| AMF | | 0.02 | 0.903 | |  | 7.26 | **0.016** |  | 0.09 | | 0.772 |  | 1.19 | 0.291 | | |  | 0.14 | 0.713 | |  | 1.5 | 0.238 | | |  |  |
| HT*AMF | | 0.52 | 0.483 | |  | 6.65 | **0.02** |  | 0 | | 0.965 |  | 2.26 | 0.152 | | |  | 0.56 | 0.463 | |  | 0.47 | 0.502 | | |  |  |

Table S8 Effects of harvest time and AMF treatment on biomass, N concentration and N content of maize in pot expt 2

^1^Values are means ± standard error, n = 5. T1 and T2, the first (day 24) and second (day 34) harvests, respectively; –AMF and +AMF, in the absence or presence of AMF, respectively; above value, significant difference between –AMF and +AMF treatments within each harvest time according to *t-*tests (*, *P* < 0.05; ***P* < 0.01; ***, *P* < 0.001); Different capital letters, significant differences between both harvest times at *P* < 0.05.

^2^ Values in bold, statistically significant sources of variation according to analysis of variance (ANOVA).

**References**

1. Jones D, Willett V. Experimental evaluation of methods to quantify dissolved organic nitrogen (DON) and dissolved organic carbon (DOC) in soil. Soil Biol Biochem. 2006;38:991-999.

2. Jakobsen I, Abbott LK, Robson AD. External hyphae of vesicular-arbuscular mycorrhizal fungi associated with *Trifolium subterraneum* L. : 1. Spread of hyphae and phosphorus inflow into roots. New Phytol. 1992;120:371-380.

3. Michotey V, Méjean V, Bonin P. Comparison of methods for quantification of cytochrome cd1-denitrifying bacteria in environmental marine samples. Appl Environ Microbiol. 2000;66:1564-1571.

4. Shifu C, Yanqing Z, Yaru C, Jia G. fastp: an ultra-fast all-in-one FASTQ preprocessor. Bioinformatics. 2018;34:884-890.

5. Magoč T, Salzberg SL. FLASH: fast length adjustment of short reads to improve genome assemblies. Bioinformatics. 2011;27:2957-2963.

6. Stackebrandt E, Goebel BM. Taxonomic note: A Place for DNA-DNA reassociation and 16S rRNA sequence analysis in the present species definition in bacteriology. Int J Syst Bacteriol. 1994;44:846-849.

7. Edgar RC. UPARSE: highly accurate OTU sequences from microbial amplicon reads. Nat Methods. 2013;10:996-998.

8. Yu J, Gu J, Wang X, Guo H, Wang J, Lei L et al. Effects of inoculation with lignocellulose-degrading microorganisms on nitrogen conversion and denitrifying bacterial community during aerobic composting. Bioresour Technol. 2020;313:123664.

9. Li D, Liu C-M, Luo R, Sadakane K, Lam TW. MEGAHIT: an ultra-fast single-node solution for large and complex metagenomics assembly via succinct de Bruijn graph. Bioinformatics. 2015;31:1674-1676.

10. Noguchi H, Park J, Takagi T. MetaGene: prokaryotic gene finding from environmental genome shotgun sequences. Nucleic Acids Res. 2006;34:5623-5630.

11. Li R, Li Y, Kristiansen K, Wang J. SOAP: short oligonucleotide alignment program. Bioinformatics. 2008;24:713-714.

12. Buchfink B, Xie C, Huson DH. Fast and sensitive protein alignment using DIAMOND. Nat Methods. 2015;12:59-60.

13. Zhang L, Feng G, Declerck S. Signal beyond nutrient, fructose, exuded by an arbuscular mycorrhizal fungus triggers phytate mineralization by a phosphate solubilizing bacterium. ISME J. 2018;12:2339-2351.

14. Livak KJ, Schmittgen TD. Analysis of relative gene expression data using real-time quantitative PCR and the 2^−ΔΔCT^ method. methods. 2001;25:402-408.

15. Koren S, Walenz BP, Berlin K. Miller JR, Bergman NH, Canu: scalable and accurate long-read assembly via adaptive k-mer weighting and repeat separation. Genome Res. 2017;27:722-736.

16. Anderson MJ. A new method for non-parametric multivariate analysis of variance. Austral Ecol. 2001;26:32-46.

17. Oksanen J, Blanchet FG, Friendly M, Kindt R, Legendre P, McGlinn D et al. Community ecology package. R package version 2.5-6. https://cran.r-project.org. Accessed 1 Sep 2019. Community ecology package. 2019.

18. Wickham H. Package, ‘ggplot2’: elegant graphics for data analysis. Springer-Verlag New York. doi, 2016, 10: 978-0.

19. Throbäck IN, Enwall K, Jarvis Å, Hallin SJF. Reassessing PCR primers targeting *nirS*, *nirK* and *nosZ* genes for community surveys of denitrifying bacteria with DGGE. FEMS Microbiol Ecol. 2004;49:401-417.

20. Henry S, Bru D, Stres B, Hallet S, Philippot LJA. Quantitative detection of the *nosZ* gene, encoding nitrous oxide reductase, and comparison of the abundances of 16S rRNA, *narG*, *nirK*, and *nosZ* genes in soils. Appl Environ Microbiol. 2006;72:5181-5189.

21. Jones CM, Graf DR, Bru D, Philippot L, Hallin SJTIj. The unaccounted yet abundant nitrous oxide-reducing microbial community: a potential nitrous oxide sink. ISME J. 2013;7:417-426.

22. Scala DJ, Kerkhof LJJFML. Nitrous oxide reductase (nosZ) gene-specific PCR primers for detection of denitrifiers and three *nosZ* genes from marine sediments. FEMS Microbiol Lett. 1998;162:61-68.

23. Usadel B. UK: John Wiley, Sons, 16S/23S rRNA sequencing. In ‘Nucleic acid techniques in bacterial systematics’. Chichester, UK: John Wiley and Sons. 1991; 115-175.
